# Novel Flurbiprofen Derivatives as Antioxidant and Anti-Inflammatory Agents: Synthesis, In Silico, and In Vitro Biological Evaluation

**DOI:** 10.3390/molecules29020385

**Published:** 2024-01-12

**Authors:** Iliyan Ivanov, Stanimir Manolov, Dimitar Bojilov, Gabriel Marc, Diyana Dimitrova, Smaranda Oniga, Ovidiu Oniga, Paraskev Nedialkov, Maria Stoyanova

**Affiliations:** 1 Department of Organic Chemistry, Faculty of Chemistry, University of Plovdiv, 24 “Tsar Assen” Street., 4000 Plovdiv, Bulgaria; bozhilov@uni-plovdiv.net (D.B.); dimitrovadiyana5@gmail.com (D.D.); marianas@uni-plovdiv.bg (M.S.); 2 Department of Pharmaceutical Chemistry, “Iuliu Hațieganu” University of Medicine and Pharmacy, 41 Victor Babeș Street, 400012 Cluj-Napoca, Romania; marc.gabriel@umfcluj.ro (G.M.); ooniga@umfcluj.ro (O.O.); 3 Department of Therapeutic Chemistry, “Iuliu Hațieganu” University of Medicine and Pharmacy, 12 Ion Creangă Street, 400010 Cluj-Napoca, Romania; smaranda.oniga@umfcluj.ro; 4 Department of Pharmacognosy, Faculty of Pharmacy, Medical University of Sofia, 2 Dunav Street, 1000 Sofia, Bulgaria; pnedialkov@pharmfac.mu-sofia.bg

**Keywords:** flurbiprofen, 2-phenethylamines, amides, synthesis, in vitro, in silico, lipophilicity, molecular docking, toxicity

## Abstract

In this study, we present the synthesis of five novel compounds by combining flurbiprofen with various substituted 2-phenethylamines. The synthesized derivatives underwent comprehensive characterization using techniques such as ^1^H- and ^13^C-NMR spectroscopy, UV-Vis spectroscopy, and high-resolution mass spectrometry (HRMS). Detailed HRMS analysis was performed for each of these newly created molecules. The biological activities of these compounds were assessed through in vitro experiments to evaluate their potential as anti-inflammatory and antioxidant agents. Furthermore, the lipophilicity of these derivatives was determined, both theoretically using the *c*Log*P* method and experimentally through partition coefficient (R_M_) measurements. To gain insights into their binding affinity, we conducted an in silico analysis of the compounds’ interactions with human serum albumin (HSA) using molecular docking studies. Our findings reveal that all of the newly synthesized compounds exhibit significant anti-inflammatory and antioxidant activities, with results statistically comparable to the reference compounds. Molecular docking studies further explain the observed in vitro results, shedding light on the molecular mechanisms behind their biological activities. Using in silico method, toxicity was calculated, resulting in LD_50_ values. Depending on the administration route, the novel flurbiprofen derivatives show lower toxicity compared to the standard flurbiprofen.

## 1. Introduction

The pursuit of novel pharmaceutical agents with enhanced therapeutic properties is a cornerstone of modern medicinal chemistry. In this context, the synthesis and evaluation of new derivatives based on established drugs provide an avenue for expanding the pharmacological landscape. This study delves into the realm of medicinal chemistry, focusing on the synthesis, in vitro biological evaluation, and molecular docking studies of a series of novel flurbiprofen amide derivatives.

Flurbiprofen, a non-steroidal anti-inflammatory drug (NSAID), has long been recognized for its anti-inflammatory and analgesic properties [1]. However, the clinical utility of flurbiprofen is often limited by its gastrointestinal side effects [2]. The strategic design of amide derivatives of flurbiprofen holds promise for attenuating these adverse effects while preserving its therapeutic benefits. Through synthetic modifications, it becomes possible to fine-tune the pharmacological profile of the parent compound, targeting specific biological pathways and optimizing drug performance.

The mode of action of flurbiprofen involves inhibiting cyclooxygenase enzymes (COX-1 and COX-2), which is reducing the synthesis of prostaglandins [3]. Prostaglandins play a key role in inflammation, pain, and fever. By decreasing their levels, flurbiprofen has anti-inflammatory, analgesic (pain-relieving), and antipyretic (fever-reducing) effects [4]. It is used to manage conditions where inflammation contributes to symptoms, such as arthritis. In the body, there are two cyclooxygenase isoenzymes: COX-1, which is consistently present and essential for maintaining gastrointestinal mucosa, kidney function, and platelet aggregation, and COX-2, which is induced during inflammation. While most NSAIDs non-selectively inhibit both COX-1 and COX-2, COX-2 selective NSAIDs such as celecoxib exclusively target COX-2, resulting in a distinct side effect profile. Notably, as COX-1 safeguards gastric mucosa and COX-2 primarily contributes to inflammation, COX-2 selective NSAIDs offer anti-inflammatory relief without compromising gastric integrity [5].

Compounds containing amide functional groups are of immense significance to both the pharmaceutical industry and human health. These chemical structures are indispensable for several compelling reasons. First and foremost, amides are biologically relevant, forming the basis of peptides, proteins, and nucleic acids [6]. They are vital components of the molecular machinery that drives various cellular processes. Understanding and manipulating amide-containing compounds is fundamental for drug development, as it allows researchers to design therapeutic agents that interact precisely with biological targets, such as enzymes, receptors, or transport proteins [7]. Amide bonds are the linchpin in drug design and development. Medicinal chemists rely heavily on these bonds to construct the structural framework of numerous pharmaceuticals. This versatile feature of amides provides meticulous control over a molecule’s three-dimensional structure, enabling the creation of compounds that can effectively engage with specific biological targets. Moreover, amide linkages contribute to improving a drug’s bioavailability. Many pharmaceutical compounds are engineered into amide-containing prodrugs to enhance their solubility, stability, and absorption within the body. This strategy significantly enhances a medication’s therapeutic potential and efficacy [8]. Amide-functionalized compounds also find applications in various pharmaceutical excipients, including binders [9], lubricants [10], and coatings for tablet formulations [11]. These excipients are crucial for producing high-quality, stable, and safe drug products, further emphasizing the pivotal role of amides in pharmaceutical manufacturing. In addition to their role in drug development, amides are essential for reducing drug toxicity. Medicinal chemists can modify the amide group to optimize a drug’s pharmacokinetics, ultimately leading to a safer and more effective medication. Furthermore, amides play a central role in the development of peptide [12] and protein [13] therapeutics, which have revolutionized the treatment of various diseases, such as diabetes and cancer. These biologics leverage amide-rich structures for their therapeutic action. Amide-containing compounds also have applications beyond traditional pharmaceuticals. They are crucial in the development of diagnostic agents for techniques such as MRI and PET scans, allowing healthcare professionals to visualize and diagnose medical conditions with precision [14,15]. Compounds containing amide functional groups are of paramount importance to the pharmaceutical industry and human health. They are integral to drug design, development, and optimization, contributing to the creation of safer, more effective medications. Their versatility extends beyond traditional small-molecule drugs and encompasses biologics and diagnostic agents, making them indispensable for improving healthcare outcomes and enhancing our understanding of human biology.

Merging flurbiprofen with various amines results in the creation of novel flurbiprofen amides. Flurbiprofen amides are bioisosteres, offering an avenue for modifying the drug’s structure to optimize its pharmacokinetics, bioavailability, and safety profile. These modifications can lead to improvements in how the drug is absorbed, metabolized, and distributed within the body, ultimately enhancing its therapeutic effectiveness. Additionally, the synthesis of flurbiprofen amides allows for targeted drug delivery. By conjugating flurbiprofen with specific amine-containing molecules, it is possible to design prodrugs that release the active compound at the desired site, potentially reducing side effects and improving patient compliance.

This study signifies a collective initiative to explore the initial potential of flurbiprofen amide derivatives as a prospective novel class of pharmaceutical agents. Considering the importance of designing novel hybrid molecules in the field of medicinal chemistry, we are actively seeking straightforward, rapid, and cost-effective approaches to prepare potential new drug candidates. The combination of synthetic, biological, and computational approaches is ready to enhance our comprehension of how structure-activity relationships govern the pharmacological effects of these compounds. 

## 2. Results and Discussion

### 2.1. Synthesis

The results of our literature search reveal a growing and robust interest in the pursuit of new derivative molecules with potential applications in the medicinal industry. The extensive body of research and publications in this field underscores the increasing recognition of the value of compounds as promising candidates for drug development. Researchers from diverse scientific disciplines are keen to explore the synergistic effects that new derivatives, aiming to unlock novel therapeutic avenues and address complex health challenges. This expanding interest not only reflects the ever-evolving nature of medicinal science but also underscores the enthusiasm and commitment of the scientific community to advance the frontiers of medical innovation [16,17,18].

**Scheme 1:**
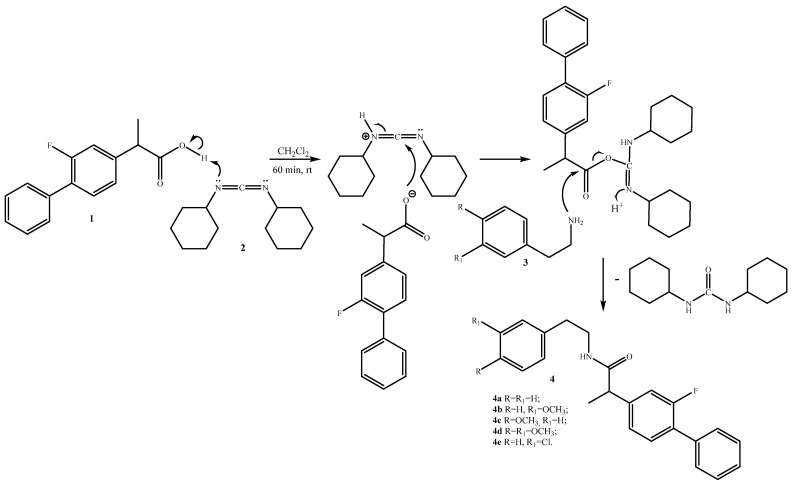
Synthesis of molecules **4a**–**e**.

In this article, we report the successful synthesis of new amide compounds starting from flurbiprofen and different substituted 2-phenethylamines (Scheme 1).

An effective and practical approach for synthesizing the desired amides from amines and carboxylic acids involves adopting the methodology outlined in the literature, employing *N*,*N*-dicyclohexylcarbodiimide **2** (Scheme 1). 

The recently obtained biofunctional molecules represent entirely new compounds (Reaxys). The latest subject to spectral characterization, which included assessing their melting points (for crystalline forms), examining their ^1^H and ^13^C NMR spectra, conducting UV and high-resolution mass spectrometry (HRMC) analysis.

### 2.2. Mass Analysis

**Scheme 2:**
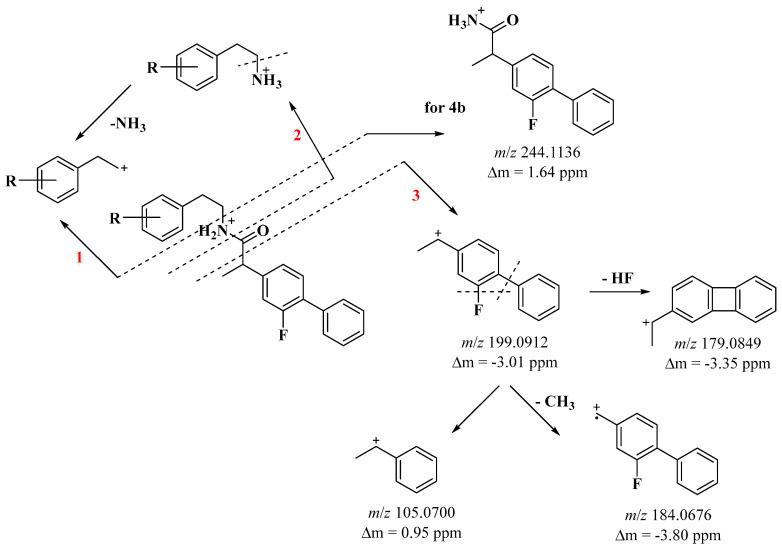
General fragmentation scheme of newly synthesized flurbiprofen derivatives **4a**–**e**.

The newly synthesized amides **4a**–**e** contain flurbiprofen in their structure. The significant difference is that in the structure of the new flurbiprofen derivatives, there is an amine. Mass spectral analysis shows that the amide bond is energetically weaker, and under ESI-MS conditions, we identified 3 pathways of molecular ion fragmentation. From the MS analysis of amides **4a**–**e**, it is evident that the cleavage of C-N bonds (path 1), N-C(O) bonds (path 2), and C(O)-C bonds (path 3) are the primary fragmentation pathways (Scheme 2).

Cleavage of the N-C bond (path 1) and N-C(O) (path 2) provided important information about the structure of the amines (*m*/*z* 122, 152, 156, 182) (Scheme 2, Figures S16–S30).

However, path 1 also results in the formation of an ion with *m*/*z* 244, which is a distinctive feature of compound **4b**. This allows for the differentiation between the two isomers, **4b** and **4c**. In compound **4b**, the methoxy group is situated in the meta position relative to the amide bond, whereas in **4c**, it is located in the para position. Additionally, the dissimilarity between the two isomers is evident in the intensity of the *m*/*z* 199 and *m*/*z* 152 fragment ions, which exhibit greater intensity in the spectrum of **4b** (see Figures S20 and S23).

Cleavage of the C(O)-C bond (pathway 3) leads to a resonance-stable aromatic cation (*m*/*z* 199) characteristic of flurbiprofen found in our previous studies (Scheme 2) [19]. In ESI-MS conditions, the identical ion experiences the removal of a neutral HF molecule, a CH_3_ radical, and a phenyl nucleus, resulting in the formation of ions with *m/z* values of 179, 184, and 105, correspondingly (as illustrated in Scheme 2 and depicted in Figures S17, S20, S23, S26 and S29).

In the case of compounds **4d** and **4e**, deviations from the typical fragmentation pattern are evident. In the amine portion of compound **4d**, which features two methoxy groups, successive loss of CH_3_ radicals is observed under ESI-MS conditions. Fragmentation of the *m/z* 165 ion leads to the formation of *m*/*z* 150 and *m*/*z* 135 ions (as depicted in Figures S26 and S27). Conversely, in the structure of amide **4e**, a chlorine atom is positioned in the meta position relative to the amide bond. The cleavage of the N-C bond (pathway 1) results in the generation of an *m/z* 139 ion, which subsequently loses a neutral HCl molecule, ultimately producing an *m/z* 103 ion (as illustrated in Figures S29 and S30).

### 2.3. In Vitro Biological Assessment

All newly synthesized flurbiprofen derivatives were tested for their in vitro antioxidant activity (HPSA), inhibition of albumin denaturation (IAD) and antitryptic activity (ATA). Table 1 presents the obtained results. 

#### 2.3.1. Hydrogen Peroxide Scavenging Activity (HPSA)

**Figure 1:**
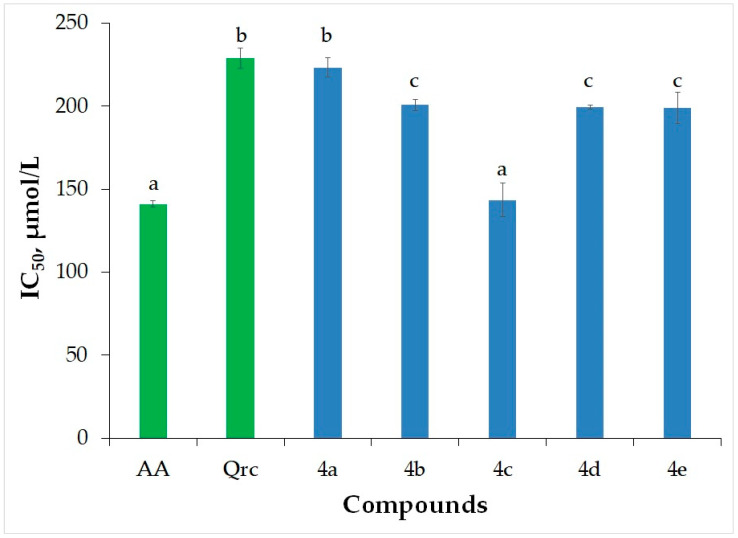
Antioxidant activity assayed as HPSA of the newly synthesized flurbiprofen derivatives. Ascorbic acid (AA) and quercetin (Qrc) were utilized as reference compounds. The results are presented as IC_50_ (µmol/L). Different letters for the same method indicate significant difference at *p* < 0.05 levels by Duncan’s test.

A free radical is a term used to describe any molecular species containing one or more unpaired electrons. Reactive oxygen species (ROS) is a comprehensive category encompassing oxygen radicals, such as superoxide, hydroxyl, peroxyl, and hydroperoxyl radicals, as well as certain non-radical oxidants such as hydrogen peroxide, hypochlorous acid, and ozone, which can easily convert into radicals themselves. ROS has been implicated in the development of sepsis. However, ROS are also naturally generated during regular metabolic processes and play roles in enzymatic reactions, mitochondrial electron transport, signal transduction, activation of nuclear transcription factors, gene expression, and the antimicrobial functions of neutrophils and macrophages. Various enzymatic and non-enzymatic mechanisms within living organisms can produce ROS, with notable sources including reactions catalyzed by the enzymes nicotinamide adenine dinucleotide phosphate (NADPH) oxidase, xanthine oxidoreductase (XOR), and myeloperoxidase (MPO) [20]. Metabolic processes primarily produce hydrogen peroxide (H_2_O_2_), which, when it interacts with iron (Fe^2+^) and copper (Cu^+^) ions, gives rise to highly reactive hydroxyl radicals (OH) through the Fenton reaction. In addition to the respiratory chain, inflammatory processes can also expedite the production of reactive oxygen species (ROS). Consequently, endogenous antioxidants play a crucial role in safeguarding the human body [21]. However, it is essential to disturb the equilibrium of the antioxidant system and incorporate external antioxidants. This, in turn, mitigates aging processes and safeguards against the disruption of the internal antioxidant system [22]. In the current study, we explored the potential of newly synthesized flurbiprofen derivatives to function as exogenous antioxidants by neutralizing H_2_O_2_. To assess their antioxidant capabilities, we compared these compounds to the natural antioxidants ascorbic acid and quercetin (Table 1). The IC_50_ values for ascorbic acid and quercetin were 141.04 µmol/L and 229.12 µmol/L, respectively. The IC_50_ values for the novel flurbiprofen derivatives (**4a**–**e**) fell within the range of 143.54 µmol/L to 223.44 µmol/L (Table 1 and Figure 1). 

The analysis of variance using Duncan’s test (with a significance level *p* < 0.05) revealed that there were not significant differences of the HPSA values between ascorbic acid and **4c**, between quercetin and **4a**, and **4b**, **4d** and **4e**. An exception to this trend is compound **4c**, which demonstrates a higher antioxidant capacity compared to both quercetin and other flurbiprofen analogs. The efficacy of these molecules can be attributed to the presence of electron-donating substituents and their positioning. In the case of compound **4c**, the methoxy group is located close to the amide group. 

Despite hydrogen peroxide’s limited reactivity, it harms cells by producing hydroxyl radicals within them [23]. The most highly reactive radicals are hydroxyl radicals, which contribute to various tissue damages associated with inflammation. Superoxide anion radicals (O_2_^•−^) and hydrogen peroxide (H_2_O_2_) within living organisms can transform into ^•^OH and ^•^O_2_, resulting in cell damage. During the inflammatory process, superoxide anion radicals are generated at the inflammation site, often accompanied by the production of other oxidizing species such as ^•^OH. In this context, the selection of new molecules plays a pivotal role. They should act as scavengers of hydroxyl radicals while also functioning as protectors by reducing inflammation and curtailing prostaglandin synthesis. Consequently, it is crucial to eliminate H_2_O_2_ to prevent the formation of ^•^OH.

#### 2.3.2. Inhibition of Albumin Denaturation (IAD)

Inflammation serves as a protective mechanism that enables the body to defend itself against infections, burns, toxic chemicals, allergens, and various other harmful stimuli. It is a vital response to injury, illness, or damage, characterized by manifestations such as heat, redness, pain, swelling, and compromised physiological functions [24].

During protein denaturation, enzymes lose their functionality since substrates can no longer bind to the active site [25]. Nonsteroidal anti-inflammatory drugs (NSAIDs) are widely prescribed medications globally due to their well-established effectiveness in alleviating pain and inflammation. NSAIDs play a crucial role in inhibiting the denaturation of proteins that function as antigens, thereby preventing the onset of autoimmune diseases [26]. These medications can result in various adverse effects, with gastric irritation being a notable cause of gastric ulcer development [27].

Protein denaturation follows an intricate mechanism characterized by alterations in electrostatic, hydrogen, hydrophobic, and disulfide bonding, exhibiting an unpredictable nature [28]. Protein denaturation can give rise to autoantigenic conditions such as rheumatoid arthritis. Consequently, inhibiting protein denaturation can effectively curb inflammatory activity [29]. Oppie [30], proposed that tissue injury during one’s lifetime could be linked to the denaturation of cellular protein components or intercellular substances. Therefore, a substance’s capacity to prevent protein denaturation signifies its potential for anti-inflammatory activity.

**Figure 2:**
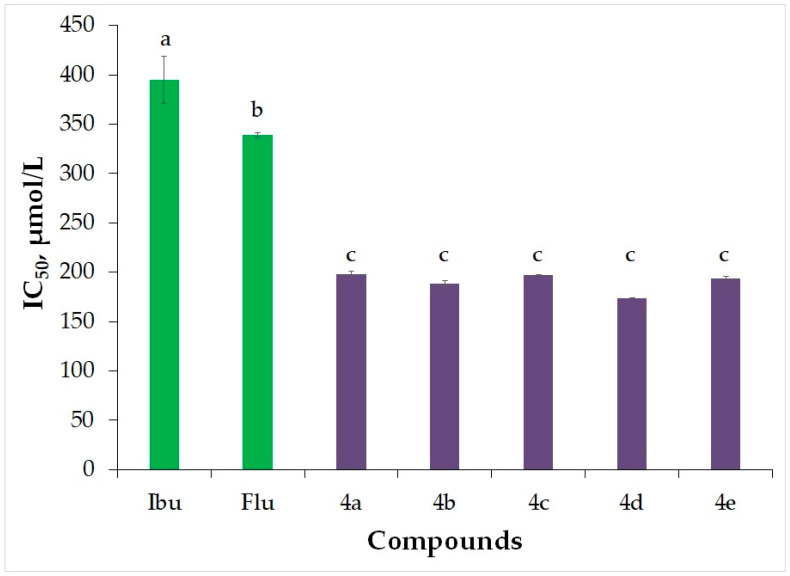
The inhibition of albumin denaturation (IAD) was assessed for the newly synthesized flurbiprofen derivatives, with ibuprofen (Ibu) and flurbiprofen (Flu) serving as reference compounds. The results for IAD are expressed as IC_50_ values (µmol/L). Different letters for the same method indicate significant difference at *p* < 0.05 levels by Duncan’s test.

In this context, we examined synthetic analogs of flurbiprofen to assess their ability to inhibit albumin denaturation. Human albumin was utilized as the subject of the study. This method allows us to gauge the extent to which albumin can be shielded from denaturation when subjected to heat. Despite flurbiprofen’s anti-inflammatory properties, the carboxyl group within its structure is responsible for the development of adverse effects, notably gastric ulcers. To mitigate this issue, the carboxyl group of flurbiprofen was altered to an amide group, employing various cyclic amines with substitutions. This modification effectively eliminates the harmful impact associated with the carboxyl group. Profen-based medications are commonly employed, and consequently, novel analogs of flurbiprofen were assessed alongside the established reference ibuprofen and flurbiprofen. The IC_50_ values for the two profens tested for inhibition of albumin denaturation (IAD) were found to be 395.08 µmol/L and 339.26 µmol/L, respectively (Table 1 and Figure 2). The obtained results revealed that the IC_50_ values for the molecules ranged from 173.74 µmol/L to 198.37 µmol/L (Table 1 and Figure 2). Analysis data indicate that the newly synthesized flurbiprofen derivatives exhibit higher activity compared to ibuprofen and flurbiprofen. The conversion of the carboxyl group to an amide and the presence of substituents such as methoxy groups and a chlorine atom in the amine structure contribute to the enhanced activity of these novel compounds. Based on these results, it can be inferred that they hold promise as reliable and potent non-steroidal anti-inflammatory drugs.

#### 2.3.3. Antitryptic Activity (ATA)

Proteinases have been identified as the root cause of arthritic disorders. Neutrophils are known to be a major source of proteinases, with several serine proteinases found in their lysosomal granules. Proteinase, which is found in leukocytes, is known to play a major part in tissue damage during inflammatory reactions, and proteinase inhibitors give a high level of protection [31,32]. In vitro anti-arthritic activity was evaluated as anti-tryptic activity [31]. 

**Figure 3:**
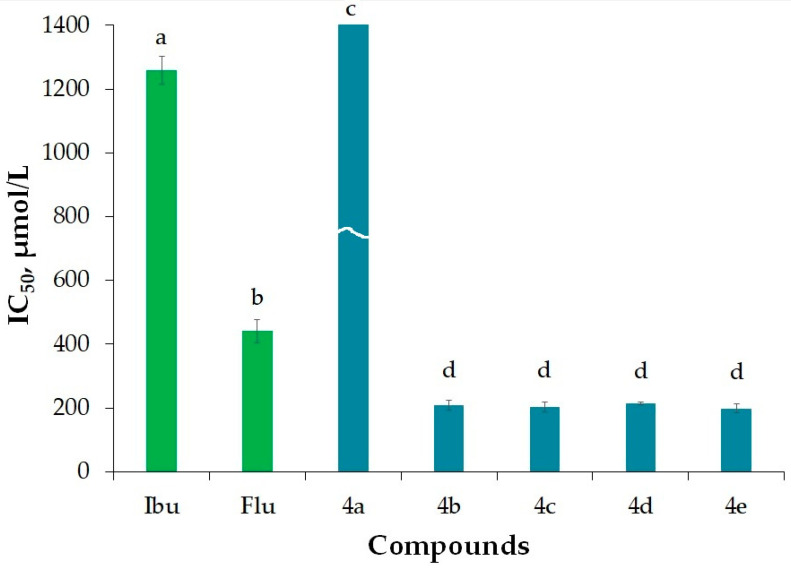
ATA of the newly synthesized flurbiprofen molecules. Ibuprofen (Ibu) and flurbiprofen (Flu) were used as reference compounds. Results for ATA are presented as IC_50_ (µmol/L). Different letters for the same method indicate significant difference at *p* < 0.05 levels by Duncan’s test.

In our in vitro study, antiarthritic activity was evaluated as antitryptic activity [31]. This research is focused on inhibiting the enzyme trypsin, which belongs to the serine proteinase group. The aim is to assess the potential of new flurbiprofen derivatives in inhibiting the enzyme’s active site. We conducted a thorough analysis of the recently synthesized flurbiprofen amides for their ATA (Table 1, Figure 3). The IC_50_ values for ATA ranged from 197.92 µmol/L to 2261.07 µmol/L. Notably, the newly created amide-flurbiprofen derivatives **4a**–**e** exhibited significantly higher activity when compared to the traditional prodrugs ibuprofen and flurbiprofen. Among these compounds, only compound **4a** displayed lower activity. This lower activity in **4a** can be attributed to the absence of substituents containing highly electronegative atoms on the benzene ring of 2-phenetylamine part, which are crucial for binding to the enzyme’s active site.

The assay results indicate that the effectiveness of the synthetic counterparts of flurbiprofen is contingent upon the presence of methoxy substituents, a relationship that has been previously established in our research [22].

#### 2.3.4. Molecular Docking

The results of the molecular docking of the enantiomers of compounds **4a**–**e** to the active site of trypsin are presented in Table 2.

Analysis of the data shows that S enantiomers have a better affinity for the active site of the trypsin compared to the R enantiomers, on all series of docking results and independent of the software used. From the entire data set, compounds (S)-**4c** and (S)-**4e** stand out, with very good affinity, regardless of the software used. On the other hand, the lowest affinity in the current series was identified for both enantiomers of **4a**, being significantly lower than for the other compounds. The lack of substitution on the benzene ring in compound **4a** leads to an obvious decreased affinity for the active site of the enzyme compared to the other compounds.

**Figure 4:**
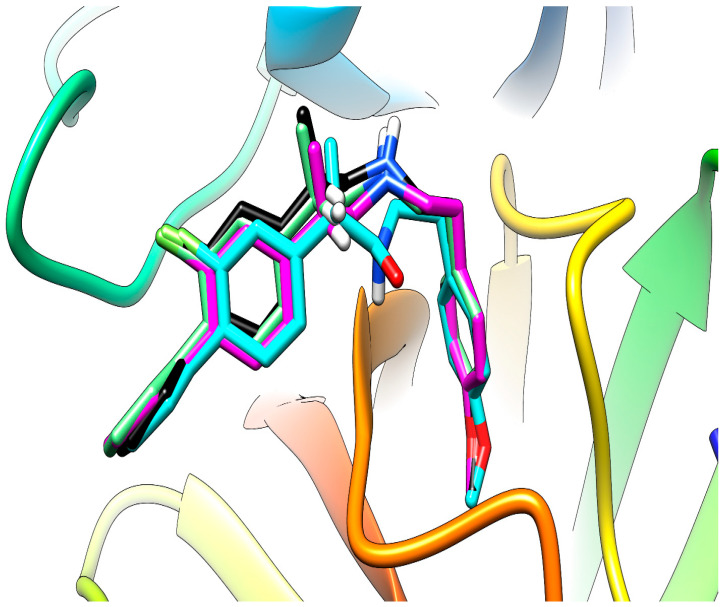
Superposition of the top conformation of (**S**)-**4c** in the active site of trypsin predicted by AutoDock (carbon atoms in cyan), AutoDock Vina rigid (carbon atoms in magenta), AutoDock Vina flexible (carbon atoms in green) and Vina-GPU (carbon atoms in black).

**Figure 5:**
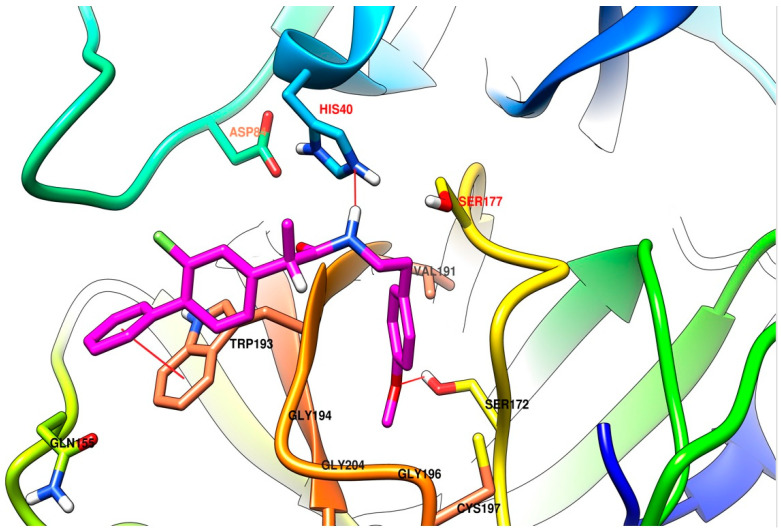
Compound (**S**)-**4c** in the active site of trypsin (labels of residues in red) predicted by AutoDock Vina in rigid mode. The compound is predicted to be involved in a π-π stacking interaction with the sidechain of Trp193, the amide bond from (**S**)-**4c** acts as a hydrogen bond donor to His40 and the methoxy-benzene moiety is accommodated in a pocket comprised of three glycine residues, a valine and a serine with which the compound is involved as a hydrogen bond acceptor. In the image are presented the tertiary elements of the protein, heavy atoms from the important amino acids and some hydrogen atoms important for the present discussion.

**Figure 6:**
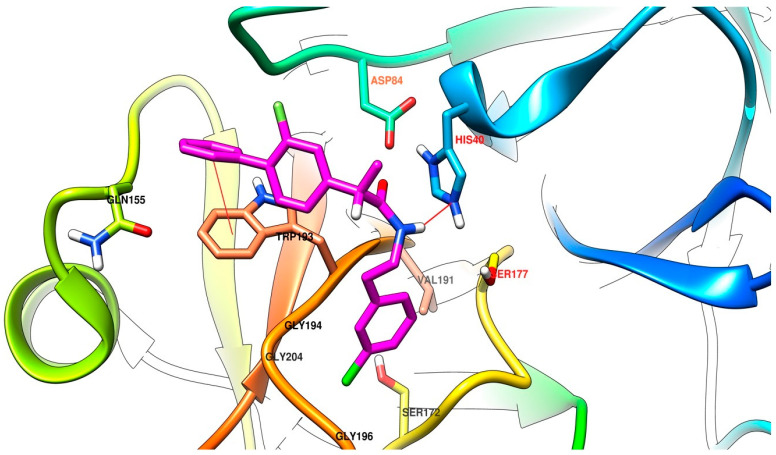
Compound (**S**)-**4e** in the active site of trypsin (labels of residues in red) predicted by AutoDock Vina in rigid mode. The compound is predicted to be involved in a π-π stacking interaction with the sidechain of Trp193, the amide bond from (**S**)-**4e** acts as a hydrogen bond donor to His40 and the meta chlorobenzene moiety is accommodated in a pocket comprised of three glycine residues, a valine and a serine. In the image are presented the tertiary elements of the protein, heavy atoms from the important amino acids and some hydrogen atoms important for the present discussion.

Comparing the results between the software used, lead to an increased affinity for the site of the enzyme in the case of the flexible mode used for AutoDock Vina, compared to the rigid mode. Anyway, the best correlation of the results is identified between the output produced by the different AutoDock Vina 1.1.2 derived software with each other, much better correlation than with AutoDock 4.2. The results of AutoDock have the best correlation from the present series with the results of AutoDock Vina in flexible mode. This is our primary use and report of Vina-GPU 1.0 and the results have a very good correlation with the output of AutoDock Vina, but the time needed for the computation was significantly lower. The superposition of the top binding conformations of compound (S)-**4c** are presented in Figure 4 for observation of the very similar results. In Figure 5 and Figure 6 are presented the top binding conformation of compounds (S)-**4c** and (S)-**4e** in the active site of trypsin, to observe the similarity in binding of the two compounds. The correlation coefficients between the four data series output from the software used are presented in Table 3.

#### 2.3.5. Experimental Determination of Lipophilicity (R_M_)

Lipophilicity plays a crucial role in the absorption of compounds, their distribution within the body, their ability to traverse various membranes and biological barriers, and their metabolism and elimination (ADME properties). Lipophilicity serves as a valuable descriptor that aids scientists in predicting and gaining deeper insights into the transportation and significance of chemical molecules within physiological and ecological systems. Its paramount significance is evident in the pharmaceutical and biotechnology industry, where it is essential for all potential drug candidates due to its pivotal role.

Lipophilicity can be assessed through either calculation or experimental methods. In our study, we determined the lipophilicity of the resulting flurbiprofen derivatives using a practical approach, employing reversed-phase thin-layer chromatography, a technique previously reported by Hadjipavlou-Litina [33]. The outcomes we acquired are presented in Table 1. 

#### 2.3.6. Prediction of Acute Rat Toxicity by Gusar Software

Prediction calculations for acute toxicity are indispensable in the synthesis of new molecules for pharmaceutical use. These calculations, often based on computational models and algorithms, play a vital role in assessing the potential adverse effects of a compound on the human body [34,35].

To assess the toxicity of recently acquired flurbiprofen derivatives, we employed the GUSAR software (https://www.way2drug.com/gusar/index.html) (Plovdiv, Bulgaria). GUSAR software was developed for constructing QSAR/QSPR models using relevant training sets. The program provides in silico predictions of LD_50_ values for rats through four administration routes: oral, intravenous, intraperitoneal, and subcutaneous.

We utilized the software to quantitatively predict in silico toxicity for LD_50_ values of the acquired flurbiprofen derivatives **4a**–**e**. The results, expressed as LD_50_ values in mg/kg, are presented in Table 4.

**Figure 7:**
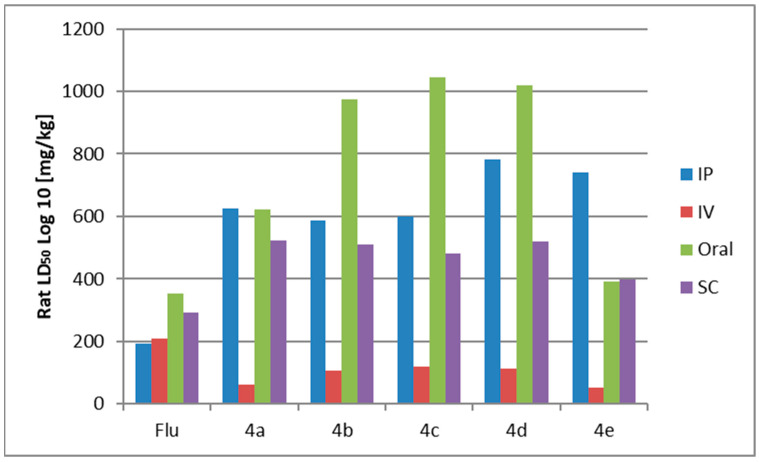
Acute rat toxicity predicted by GUSAR.

Observing the obtained data (Table 4, Figure 7), it can be seen that all compounds **4a–e** show high calculated toxicity when administrated intravenously (IV). When administered intravenously, Compound **4e** exhibits four times greater toxicity than flurbiprofen. The existence of methoxy groups in the benzene core, whether one or two, results in a lower level of toxicity compared to compounds **4a**, which lacks substituents in the benzene core, and compound **4e**, which features a chlorine atom as a substituent in the benzene core. When comparing the four administration routes provided, it becomes evident that the average lethal doses of the newly synthesized molecules, representing the amount needed to cause the demise of 50% of a given test population, are considerably smaller when administered intravenously. This can be explained by the direct introduction of the substance into the bloodstream, resulting in a bioavailability of typically 100%, thereby ensuring that the entire dose reaches systemic circulation. The inclusion of two methoxy groups in the phenyl nucleus renders compound **4d** the least toxic among the derivative compounds, including the standard flurbiprofen, when administered via the intraperitoneal (IP) route. The derivatives are arranged in the following order of decreasing toxicity by the IP route: **Flu** > **4b** > **4c** > **4a** > **4e** > **4d**. 

In terms of oral administration, it can be deduced that the inclusion of methoxyl groups (**4b**–**d**) significantly diminishes toxicity compared to flurbiprofen, specifically by a factor of three or more (Table 4, Figure 7). The study results suggest that administering this drug family orally is more favorable, resulting in a reduction of toxic effects.

## 3. Materials and Methods

### 3.1. General

All reagents and chemicals were procured from commercial suppliers, namely Sigma-Aldrich S.A. and Riedel-de Haën, Sofia, Bulgaria, and used without further purification. NMR spectral data were acquired using a Bruker Avance Neo 400 spectrometer (BAS-IOCCP–Sofia, Bruker, Billerica, MA, USA). The ^1^H-NMR and ^13^C-NMR spectra for all compounds were obtained in DMSO-*d*_6_ at 400 MHz and 101 MHz, respectively. Chemical shifts are reported in relative ppm and were calibrated with respect to tetramethylsilane (TMS) at δ = 0.00 ppm, serving as the internal standard, with coupling constants denoted in Hz. NMR spectra were recorded at room temperature (approximately 295 K). For mass spectrometry (MS) analysis, a Q Exactive Plus high-resolution mass spectrometer (HRMS) with a heated electrospray ionization source (HESI-II) from Thermo Fisher Scientific, Inc., Bremen, Germany, was employed. The MS system was equipped with a Dionex Ultimate 3000RSLC ultrahigh-performance liquid chromatography (UHPLC) system from Thermo Fisher Scientific, Inc., Waltham, MA, USA. Thin-layer chromatography (TLC) was conducted using 0.2 mm Fluka silica gel 60 plates obtained from Merck KGaA, Darmstadt, Germany.

### 3.2. Synthesis

To flurbiprofen (1 mmol, 0.244 g) dissolved in CH_2_Cl_2_, *N*,*N*-dicyclohexylcarbodiimide (1 mmol, 0.206 g) is added, and the reaction mixture was stirred on a magnetic stirrer for 10 min at room temperature. To the resulting reaction mixture, the respective amine (1 mmol) was added, and the reaction mixture was stirred for an additional 50 min. After the reaction was complete, the reaction mixture was filtered through a sintered glass filter to separate the *N*,*N*-Dicyclohexylurea. The resulting filtrate is sequentially washed with a diluted solution of hydrochloric acid (HCl:H_2_O = 1:4), sodium carbonate, and brine, followed by drying with anhydrous Na_2_SO_4_ and concentration. After removing the CH_2_Cl_2_ by distillation, the resulted amide was dissolved in ethyl acetate. The flask was placed in an ice bath for 30 min until complete crystallization of dicyclohexylurea. Subsequent filtration through a sintered glass filter helps remove the by-product of the reaction–dicyclohexylurea. The filtrate was distilled using a rotary vacuum evaporator to remove the ethyl acetate. The obtained amide was dissolved in CH_2_Cl_2_ and sequentially washed with a solution of sodium carbonate and water. It was then dried with anhydrous Na_2_SO_4_ and concentrated.

**4a** 2-(2-fluoro-[1,1′-biphenyl]-4-yl)-N-phenethylpropanamide

White crystals (m.p. 67–68 °C), yield 96% (0.333 g), ^1^H NMR (600 MHz, CDCl_3_) δ 7.56–7.52 ppm (m, 2H), 7.47–7.43 ppm (m, 2H), 7.40–7.34 ppm (m, 2H), 7.25–7.16 ppm (m, 3H), 7.09–7.01 ppm (m, 4H), 5.49 ppm (s, 1H), 3.58–3.48 ppm (m, 2H), 3.43 ppm (dtd, *J* = 13.6, 6.9, 5.7 Hz, 1H), 2.75 ppm (t, *J* = 7.1 Hz, 2H), 1.52 ppm (d, *J* = 7.2 Hz, 3H). ^13^C NMR (151 MHz, CDCl_3_) δ 173.4 ppm (C=O), 159.8 ppm (d, ^1^*J*_C-F_ = 249.0 Hz), 142.6 ppm (Ar), 142.6 ppm (Ar), 138.7 ppm (Ar), 135.4 ppm (Ar), 131.1 ppm (d, ^3^*J*_C-F_ = 3.8 Hz), 128.9 ppm (Ar), 128.8 ppm (Ar), 128.6 ppm (Ar), 128.5 ppm (Ar), 128.5 ppm (Ar), 127.8 ppm (Ar), 126.5 ppm (Ar), 123.6 ppm (Ar), 123.6 ppm (Ar), 115.4 ppm (Ar), 115.3 ppm (d, ^2^*J*_C-F_ = 23.3 Hz), 46.6 ppm (COCHCH3), 40.8 ppm (CH_2_CH_2_NH), 35.5 ppm (ArCH_2_CH_2_), 18.3 ppm (CHCH_3_). UV λ*_max_*, MeOH: 270 (ε = 19,700) nm. HRMS Electrospray ionization (ESI) *m*/*z* calcd for [M + H]^+^ C_23_H_23_FNO^+^ = 348.1758, found 348.1748 (mass error Δm = −2.87 ppm).

**4b** 2-(2-fluoro-[1,1′-biphenyl]-4-yl)-N-(3-methoxyphenethyl)propanamide

White Oil, yield 98% (0.369 g), ^1^H NMR (600 MHz, CDCl_3_) δ 7.48–7.42 ppm (m, 2H), 7.33 ppm (dt, *J* = 45.5, 7.7 Hz, 4H), 7.09–7.03 ppm (m, 1H), 7.02–6.94 ppm (m, 2H), 6.64 ppm (dd, *J* = 8.2, 2.6 Hz, 1H), 6.56–6.52 ppm (m, 2H), 5.41 ppm (s, 1H), 3.64 ppm (s, 3H), 3.48–3.41 ppm (m, 2H), 3.36 ppm (dq, *J* = 13.2, 6.7 Hz, 1H), 2.65 ppm (t, *J* = 6.9 Hz, 2H), 1.44 ppm (d, *J* = 7.2 Hz, 3H). ^13^C NMR (151 MHz, CDCl_3_) δ 173.4 ppm (C=O), 159.8 ppm (C(Ar)OCH_3_), 159.8 ppm (d, ^1^*J*_C-F_ = 249.1 Hz), 140.3 ppm (Ar), 135.4 ppm (Ar), 131.1 ppm (Ar), 131.06 ppm (d, ^3^*J*_C-F_ = 3.9 Hz), 131.05 ppm (Ar), 129.6 ppm (Ar), 129.0 ppm (Ar), 128.9 ppm (Ar), 128.5 ppm (Ar), 127.7 ppm (Ar), 123.6 ppm (Ar), 123.6 ppm (Ar), 121.1 ppm (Ar), 115.4 ppm (Ar), 115.3 ppm (d, ^2^*J*_C-F_ = 23.3 Hz), 115.2 ppm (Ar), 114.5 ppm (Ar), 111.7 ppm (Ar), 55.1 ppm (ArOCH_3_), 46.7 ppm (COCHCH_3_), 40.6 ppm (CH_2_CH_2_NH), 35.5 ppm (ArCH_2_CH_2_), 18.4 ppm (CHCH_3_). UV *λ*_max_, MeOH: 270 (*ε* = 22,900) nm. HRMS Electrospray ionization (ESI) *m*/*z* calcd for [M + H]^+^ C_24_H_25_FNO_2_^+^ = 378.1864, found 378.1855 (mass error Δm = −2.38 ppm).

**4c** 2-(2-fluoro-[1,1′-biphenyl]-4-yl)-N-(4-methoxyphenethyl)propanamide

White crystals (m.p. 98–99 °C), yield 96% (0.370 g), ^1^H NMR (600 MHz, CDCl_3_) δ 7.46 ppm (ddt, *J* = 13.8, 8.1, 1.5 Hz, 2H), 7.37 ppm (dd, *J* = 8.5, 7.0 Hz, 2H), 7.33–7.26 ppm (m, 2H), 7.07–6.91 ppm (m, 2H), 6.89–6.84 ppm (m, 2H), 6.71–6.65 ppm (m, 2H), 5.35 ppm (s, 1H), 3.63 ppm (s, 3H), 3.42 ppm (dq, *J* = 12.9, 6.9, 6.5 Hz, 2H), 3.31 ppm (dtd, *J* = 13.6, 6.8, 5.6 Hz, 1H), 2.61 ppm (t, *J* = 6.8 Hz, 2H), 1.44 ppm (d, *J* = 7.2 Hz, 3H). ^13^C NMR (151 MHz, CDCl_3_) δ 173.3 ppm (C=O), 159.8 ppm (d, ^1^*J*_C-F_ = 249.1 Hz), 158.9 ppm (Ar), 158.2 ppm (Ar), 135.4 ppm (Ar), 131.1 ppm (d, ^3^*J*_C-F_ = 4.1 Hz), 130.6 ppm (Ar), 129.7 ppm (Ar), 128.95 ppm (Ar), 128.93 ppm (Ar), 128.53 ppm (Ar), 128.51 ppm (Ar), 127.8 ppm (Ar), 123.8 ppm (Ar), 123.7 ppm (Ar), 115.4 ppm (d, ^2^*J*_C-F_ = 23.3 Hz), 114.0 ppm (Ar), 55.2 ppm (ArOCH_3_), 46.7 ppm (COCHCH_3_), 40.9 ppm (CH_2_CH_2_NH), 34.6 ppm (ArCH_2_CH_2_), 18.3 ppm (CHCH_3_). UV *λ*_max_, MeOH: 252 (*ε* = 18,100) nm, 270 (*ε* = 20,400) nm. HRMS Electrospray ionization (ESI) *m*/*z* calcd for [M + H]^+^ C_24_H_25_FNO_2_^+^ = 378.1864, found 378.1854 (mass error Δm = −2.64 ppm).

**4d** N-(3,4-dimethoxyphenethyl)-2-(2-fluoro-[1,1′-biphenyl]-4-yl)propanamide

White crystals (m.p. 94–95 °C), yield 99% (0.403 g), ^1^H NMR (600 MHz, DMSO) δ 7.58 ppm (s, 1H), 7.41–7.34 ppm (m, 2H), 7.31–7.20 ppm (m, 4H), 7.04–7.00 ppm (m, 2H), 6.64 ppm (d, *J* = 8.1 Hz, 1H), 6.60 ppm (d, *J* = 2.1 Hz, 1H), 6.49 ppm (dd, *J* = 8.1, 2.1 Hz, 1H), 3.55 ppm (s, 3H), 3.54 ppm (s, 3H), 3.49 ppm (q, *J* = 7.1 Hz, 1H), 3.18–3.09 ppm (m, 2H), 2.50 ppm (t, *J* = 7.1 Hz, 2H), 1.21 ppm (d, *J* = 7.1 Hz, 3H). ^13^C NMR (151 MHz, DMSO) δ 173.1 ppm (C=O), 159.4 ppm (d, ^1^*J*_C-F_ = 246.0 Hz), 149.6 ppm (Ar), 148.2 ppm (Ar), 144.8 ppm (Ar), 135.6 ppm (Ar), 132.8 ppm (Ar), 130.7 ppm (Ar), 129.1 ppm (d, ^3^*J*_C-F_ = 3.1 Hz), 128.9 ppm (Ar), 128.0 ppm (Ar), 124.2 ppm (Ar), 124.2 ppm (Ar), 121.3 ppm (Ar), 115.3 ppm (d, ^2^*J*_C-F_ = 23.2 Hz), 114.0 ppm (Ar), 113.4 ppm (Ar), 56.5 ppm (OCH_3_), 56.3 ppm (OCH_3_), 48.2 ppm (COCHCH_3_), 45.3 ppm (CH_2_CH_2_NH), 35.1 ppm (ArCH_2_CH_2_), 18.8 ppm (CHCH_3_). UV *λ*_max_, MeOH: 265 (*ε* = 19,500) nm. HRMS Electrospray ionization (ESI) *m*/*z* calcd for [M + H]^+^ C_25_H_27_FNO_3_^+^ = 408.1969, found 408.1961 (mass error Δm = −1.96 ppm), calcd for [M + Na]^+^ C_25_H_27_FNO_3_Na^+^ = 430.1789, found 430.1780 (mass error Δm = −2.09 ppm).

**4e** N-(3-chlorophenethyl)-2-(2-fluoro-[1,1′-biphenyl]-4-yl)propanamide

White Oil, yield 98% (0.377 g), ^1^H NMR (600 MHz, CDCl_3_) δ 7.50–7.43 ppm (m, 2H), 7.42–7.35 ppm (m, 2H), 7.33–7.27 ppm (m, 2H), 7.12–7.04 ppm (m, 2H), 7.02–6.97 ppm (m, 3H), 6.84 ppm (dt, *J* = 6.9, 1.7 Hz, 1H), 5.40 ppm (s, 1H), 3.50–3.39 ppm (m, 2H), 3.33 ppm (dtd, *J* = 13.7, 6.9, 5.7 Hz, 1H), 2.67 ppm (t, *J* = 6.8 Hz, 2H), 1.45 ppm (d, *J* = 7.2 Hz, 3H). ^13^C NMR (151 MHz, CDCl_3_) δ 173.5 ppm (C=O), 159.8 ppm (d, ^1^*J*_C-F_ = 249.1 Hz), 142.5 ppm (Ar), 142.5 ppm (Ar), 140.8 ppm (Ar), 135.4 ppm (Ar), 134.3 ppm (Ar), 131.1 ppm (d, ^3^*J*_C-F_ = 4.1 Hz), 129.8 ppm (Ar), 129.0 ppm (Ar), 128.9 ppm (Ar), 128.53 ppm (Ar), 128.51 ppm (Ar), 127.8 ppm (Ar), 127.0 ppm (Ar), 126.7 ppm (Ar), 123.62 ppm (Ar), 123.60 ppm (Ar), 115.4 ppm (Ar), 115.3 ppm (d, ^2^*J*_C-F_ = 23.3 Hz), 46.6 ppm (COCHCH_3_), 40.5 ppm (CH_2_CH_2_NH), 35.2 ppm (ArCH_2_CH_2_), 18.4 ppm (CHCH_3_). UV *λ*_max_, MeOH: 270 (*ε* = 12,000) nm. HRMS Electrospray ionization (ESI) *m*/*z* calcd for [M + H]^+^ C_23_H_22_ClFNO^+^ = 382.1368, found 382.1363 (mass error Δm = −1.31 ppm).

### 3.3. HRMS Analysis

The HESI source operated in a positive ionization mode under the following conditions: a spray voltage of +3.5 kV, a capillary and probe heater temperature of 320 °C, sheath gas flow rate set at 36 arbitrary units (a.u.), auxiliary gas flow rate at 11 a.u., spare gas flow rate at 1 a.u. (a.u. denoting values configured by the Exactive Tune software), and an S-Lens RF level of 50.00. Nitrogen was employed for both sample nebulization and collision gas within the HCD cell. For mass spectrometry analysis, 1 µL aliquots of sample solutions (approximately 20 µg mL^−1^) were introduced into the mass spectrometer via the UHPLC system. Each chromatographic run was conducted isocratic using a mobile phase consisting of a mixture of water, acetonitrile, methanol, and acetic acid in a ratio of 25:50:25:0.2 with a solvent flow at a rate of 300 μL min^−1^. The MS experiment employed was Full MS–ddMS^2^ (Top 5). In the full scan MS, the instrument settings were as follows: a resolution of 70,000 (at *m*/*z* 200), automatic gain control (AGC) target of 3 × 10^6^, maximum injection time (IT) of 100 ms, and a mass range of *m*/*z* 100–500. For the ddMS^2^ scans, the parameters included a resolution of 17,500 (at *m*/*z* 200), AGC target of 1 × 10^5^, maximum IT of 50 ms, loop count of 5, isolation window of 2.0 *m*/*z*, and stepped normalized collision energy (NCE) set to 10, 20, and 60. Data-dependent (dd) settings comprised a maximum AGC target of 5 × 10^4^, dynamic exclusion set at 1 s, and the use of preferred peptide match and isotope exclusion. Data acquisition and processing were conducted using Xcalibur software (Thermo Fisher Scientific, Waltham, MA, USA) version 4.0.

### 3.4. Biological Activity

#### 3.4.1. Hydrogen Peroxide Scavenging Activity (HPSA) 

The Manolov et al. method was used to assess the hydrogen peroxide scavenging capability [22]. A 43 mM solution of H_2_O_2_ was prepared in potassium phosphate buffer solution (0.2 M, pH 7.4). The analysis of the samples was carried out as follows: in test tubes, 0.6 mL H_2_O_2_ (43 mM), 1 mL sample or reference compounds with different concentrations (20–1000 µg/mL), and 2.4 mL potassium phosphate buffer solution were mixed. The mixture was stirred and incubated in the dark for 10 min at 37 °C. Absorbance was measured at 230 nm with a spectrophotometer (Camspec M508, Leeds, UK) against a blank solution containing phosphate buffer and H_2_O_2_ without the sample. Ascorbic acid and quercetin were used as reference compounds. The percentage HPSA of the samples was evaluated by comparing with a blank sample and calculated using the following formula:

$$I,\%(HPSA)=\left[\frac{A_{blank}-(A_{TS}-A_{CS})}{A_{blank}}\right]\times 100$$

where *A_blank_* is the absorbance of the blank sample, *A_CS_* is the absorbance of the control sample, and *A_TS_* is the absorbance of the test sample.

#### 3.4.2. Inhibition of Albumin Denaturation (IAD)

In vitro analysis of anti-inflammatory activity was assessed as inhibition of albumin denaturation (IAD). The analysis was performed according Manolov et al. method [22]. The experiment was performed with human albumin. The solution of albumin (1%) was prepared in distilled water (pH 7.4). The tested and the reference compounds were dissolved firstly in PBS resulting in 1000 μg/mL stock solutions. Then a series of working solutions with different concentrations (20–500 μg/mL) in PBS were prepared. The reaction mixture was containing 2 mL test sample/reference of different concentrations and 1 mL albumin (1%). The mixture was incubated at 37 °C for 15 min and then heated at 70 °C for 15 min in water bath. After cooling, the turbidity was measured at 660 nm with a spectrophotometer (Camspec M508, Leeds, UK). Ibuprofen and flurbiprofen were used as reference compounds. The experiment was performed three times. Percentage inhibition of albumin denaturation (IAD) was calculated against the control. The control sample is albumin with the same concentration dissolved in distilled water.

$$\%IAD=\left[\frac{A_{blank}-A_{sample}}{A_{blank}}\right]\times 100$$

#### 3.4.3. Antitryptic Activity (ATA) 

This method is known also as an in vitro anti-arthritic activity. The analysis was performed according to the method of Oyedapo and Femurewa [31] with minor modification as described by Manolov et al. [22]. The reaction mixture was containing 2 mL 0.06 mg/mL bovine trypsin, 1 mL Tris–HCl buffer (20 mM, pH 7.4) and 1 mL test sample or reference (in methanol) of different concentrations (20–1000 μg/mL). The mixture was incubated at 37 °C for 5 min. Then 1 mL of human albumin (4% *v*/*v*) was added. The mixture was incubated for an additional 20 min. To the mixture 2 mL of 70% perchloric acid was added for termination of the reaction. The cloudy suspension was cooled and centrifuged at 5000 rpm for 20 min. The absorbance of the supernatant was measured at 280 nm with a spectrophotometer (Camspec M508, Leeds, UK) against control solution. The control solution was sample or reference in methanol with different concentrations. Ibuprofen and flurbiprofen were used as reference compounds. The analysis was performed three times. The percentage of antitryptic activity (ATA) of the samples was evaluated by comparing with a blank sample. The blank sample is prepared as the test sample but with a small exception–perchloric acid is added before albumin.

$$\%ATA=\left[\frac{A_{blank}-\left(A_{TS}-A_{CS}\right)}{A_{blank}}\right]\times 100$$

where *A_blank_* is the absorbance of the blank sample, *A_CS_* is the absorbance of the control solution (test sample in different concentrations) and *A_TS_* is the absorbance of the test samples.

#### 3.4.4. Molecular Docking

The molecular docking of the ligands was performed using AutoDock 4.2, AutoDock Vina 1.1.2 and Vina-GPU 1.0 [36,37,38] against the bovine trypsin (PDB: 3AAS, resolution 1.75 Å) [39,40]. The choice of this structure was made so that the protein target was of bovine origin, as used in the in vitro experiment, the age of the deposited structure, the score and the resolution. Three software were selected for molecular docking since, although their names are similar, the principle on which they operate is different. Since it is known that molecular docking studies can lead to false-positive results, running more software that are based on different principles can be used to cross-validate the results. AutoDock uses a Lamarckian Genetic Algorithm for global optimization and an empirical scoring function, while AutoDock Vina uses a different local search optimization algorithm and an improved scoring function [38,41,42,43,44]. Vina-GPU, as the name indicates, uses graphics processing units (GPUs) to speed up docking calculations. The software is specifically optimized for NVIDIA GPUs and uses their parallel processing capabilities to perform docking simulations much faster than the CPU based version [36].

The automatic clustering analysis from AutoDock was used and the results were filtered according to the most populated cluster (2 Å root mean square deviation of conformations coordinates), to confirm that the best binding pose is one of the most found poses from the total of poses generated.

AutoDock Vina was used in two ways, in one the protein was considered rigid and in the second the amino acid residues from the active site of trypsin were set to be flexible (His40, Asp84, Ser177), according to the results of a BLAST analysis of FASTA sequence of the protein using the blastp suite https://blast.ncbi.nlm.nih.gov (accessed on 9 September 2023) [45]. The search space was set as cube, with sides equal to 20 for ADV and 54 for AD (spacing = 0.375). The cartesian coordinates were set to x = 0.140, y = 14.007, z = 18.625, to include the three specified residues.

For each compound, two ligand files were created, one for each R and S isomer using Avogadro 1.2.0 and were prepared following the previously reported protocol using AutoDockTools 1.5 [37,46,47,48]. AutoDock was requested to generate 200 poses for each ligand (a high number to ensure a sufficiently large sample of conformations to be able to do the clustering analysis to select the top binding conformation from the most populated cluster), AutoDock Vina was requested to generate 20 poses and for and Vina-GPU the parameters were set to thread = 8000 and search depth = 1000.

The preparation of the macromolecule as a target followed the standard procedure already reported by our group–removal of the co-crystallized molecules, addition of the polar hydrogen atoms and addition of charges [49]. Redocking of the co-crystallized ligand could not be performed since its structure is composed of an organic ligand that chelates a copper ion coordinatively linked to the imidazole of His40 of the protein, being impossible to perform the molecular docking using the available software. To overcome this, multi-software docking was performed to cross-validate the results reported in the present paper.

The visual analysis of the results of the molecular docking study was performed using Chimera 1.10.2 [50].

#### 3.4.5. Determination of Lipophilicity as *c*Log*P*

The lipophilicity of the compounds was calculated using the software: ACD/ChemSketch/LogP Predictor v.14.08.

#### 3.4.6. Experimental Determination of Lipophilicity (R_M_) 

The method employed for assessing the lipophilicity of flurbiprofen derivatives followed the procedure outlined by Hadjipavlou-Litina [33].

#### 3.4.7. Statistical Analysis

All of the analyses were made in triplicates. Data were expressed as mean ± SD. The level of significance was set at *p* < 0.05. The mean IC_50_ value was estimated based on three replicates by interpolating the graphical dependence of activity on concentration. Statistical program SPSS 19.0 software was used for data analysis by one-way ANOVA followed by Duncan’s post hoc test to evaluate differences between mean values of activities (SPSS Inc., Chicago, IL, USA).

## 4. Conclusions

The objective of this research is to create derivatives of flurbiprofen that have the potential to be biologically active while minimizing adverse effects on human health. Flurbiprofen was subjected to derivatization with a variety of amines, resulting in the generation of new flurbiprofen derivatives distinguished by their hydrogen peroxide scavenging activity (HPSA). Our investigation revealed that these novel compounds exhibit dual properties, displaying both antioxidant activity and in vitro anti-inflammatory activity, as assessed by IAD. Significant insights can be derived from this. Thanks to their favorable lipophilic properties, these novel derivatives possess the capacity to combat ROS effectively, preventing lipid peroxidation and safeguarding the cell membrane against the detrimental effects of ROS. The recently developed compounds demonstrate superior efficacy compared to flurbiprofen, thereby enhancing their potential to inhibit albumin denaturation. Given their high activity, they may also possess the capability to suppress prostaglandin production. These amides not only inherit the attributes of profens but also exhibit greater activity than them. In relation to ATA, derivatives 4**b**–**e** displayed markedly elevated activity compared to profens. This finding is further substantiated by the conducted molecular docking analysis, which specifically revealed that the S-enantiomers exhibit a stronger binding affinity for trypsin’s active site. The research indicates that these compounds could hold significant potential for pharmacological applications. Compounds **4a**–**e** display high intravenous toxicity, with **4e** four times more toxic than flurbiprofen. Methoxy groups, especially in **4b**–**d**, are associated with lower toxicity. Intravenous administration yields smaller lethal doses due to 100% bioavailability. Compound **4d** is the least toxic intraperitoneally (order: **Flu** > **4b** > **4c** > **4a** > **4e** > **4d**). Orally, **4b**–**d** significantly reduce toxicity compared to flurbiprofen by a factor of three or more, suggesting oral administration is more favorable, minimizing toxic effects. This positions them as promising and captivating candidates for future biological evaluation.

## Figures and Tables

**Table 1:** The in vitro outcomes of the performed biological assays are summarized here. The results for antioxidant activity (HPSA), inhibition of albumin denaturation (IAD), and antitryptic activity (ATA) are expressed in terms of IC_50_ values. Ascorbic acid (AA), quercetin (Qrc), ibuprofen (Ibu), and flurbiprofen (Flu) were used as reference compounds. R_M_, denoting lipophilicity, is a dimensionless measure derived from thin-layer chromatography and is dependent on the R_f_ value.

Compounds	HPSA	IAD	ATA	R_M_ ± SD	*c*Log*P*
	IC_50_ ± SD (µmol/L)				
**AA**	141.0 ± 2.0	-	-	-	
**Qrc**	229 ± 6	-	-	-	
**Ibu**	618 ± 25	395 ± 24	1260 ± 44	1.11 ± 0.01	3.72
**Flu**	1158 ± 64	339.3 ± 2.3	441 ± 36	1.37 ± 0.02	3.57
**4a**	223 ± 6	198.4 ± 2.3	2261 ± 73	1.24 ± 0.02	4.95
**4b**	200.8± 3.3	188.3 ± 3.0	208 ± 15	1.13 ± 0.02	5.04
**4c**	144 ± 10	196.9 ± 0.5	203 ± 14	1.21 ± 0.02	5.07
**4d**	199.5 ± 1.3	173.74 ± 0.13	215 ± 5	1.33 ± 0.02	4.90
**4e**	199 ± 9	193.5 ± 2.2	198 ± 14	1.41 ± 0.03	5.44

**Table 2:** The results of the docking study made using AutoDock 4.2, AutoDock Vina 1.1.2 in rigid and flexible mode and Vina-GPU 1.0 expressed as binding affinity of the studied compounds to the active site of trypsin expressed as variation of the Gibbs free energy (ΔG kcal/mol).

Compound	Enantiomer	AutoDock 4.2	AutoDock Vina 1.1.2 Rigid	AutoDock Vina 1.1.2 Flexible	Vina-GPU 1.0
**4a**	R	−6.7	−7.4	−7.5	−7.3
	S	−6.9	−7.7	−7.7	−7.5
**4b**	R	−7.3	−7.6	−7.6	−7.6
	S	−7.4	−8.0	−8.0	−8.0
**4c**	R	−7.5	−8.1	−8.3	−8.0
	S	−7.8	−8.2	−8.5	−8.2
**4d**	R	−7.4	−7.5	−7.7	−7.3
	S	−7.5	−7.9	−7.9	−7.9
**4e**	R	−7.6	−7.8	−8.0	−7.7
	S	−7.7	−8.0	−8.3	−8.0

**Table 3:** The correlation coefficient (R) between the series ΔG resulted from the molecular docking study made using AutoDock 4.2, AutoDock Vina 1.1.2 in rigid and flexible mode and Vina-GPU 1.0.

	AutoDock 4.2	AutoDock Vina 1.1.2 Rigid	AutoDock Vina 1.1.2 Flexible	Vina-GPU 1.0
**AutoDock 4.2**	-	0.75	0.82	0.76
**AutoDock Vina 1.1.2 rigid**	0.75	-	0.94	0.98
**AutoDock Vina 1.1.2 flexible**	0.82	0.94	-	0.90
**Vina-GPU 1.0**	0.76	0.98	0.90	-

**Table 4:** Predicted acute toxicity in rats using GUSAR software.

	Rat LD_50_ Log10 (mg/kg)			
	IP	IV	Oral	SC
**Flu**	191.5 ^4a^	209.9 ^4a^	353.1 ^4a^	293.5 ^4a^
**4a**	626.3 ^5a^	62.29 ^4a^	621.3 ^4a^	521.3 ^4a^
**4b**	587.5 ^5a^	106.2 ^4a^	973.6 ^4a^	511.1 ^4a^
**4c**	598.4 ^5a^	119.5 ^4a^	1045 ^4a^	479.9 ^4a^
**4d**	781.4 ^5a^	114.2 ^4a^	1020 ^4a^	521.1 ^4a^
**4e**	739.3 ^5a^	51.4 ^4a^	392.4 ^4a^	398.5 ^4a^

IP-intraperitoneal, IV-intravenous, SC-subcutaneous, Applicability domain: a-compound falls in the applicability domain of models; b-compound does not fall in the applicability domain of models. The superscript numbers show the acute rodent toxicity classification by OECD (The Organization for Economic Co-operation and Development). OECD classifies the chemicals into five categories, where 1 is highly toxic and 5 is practically non-toxic.

## Data Availability

Data are contained within the article and Supplementary Materials.

## Supplementary Materials

The following supporting information can be downloaded at: https://www.mdpi.com/article/10.3390/molecules29020385/s1, Figure S1: ^1^H-NMR spectrum of compound **4a**; Figure S2: ^1^H-NMR spectrum of compound **4b**; Figure S3: ^1^H-NMR spectrum of compound **4c**; Figure S4: ^1^H-NMR spectrum of compound **4d**; Figure S5. ^1^H-NMR spectrum of compound **4e**; Figure S6: ^13^C-NMR spectrum of compound **4a**; Figure S7: ^13^C-NMR spectrum of compound **4b**; Figure S8: ^13^C-NMR spectrum of compound **4c**; Figure S9: ^13^C-NMR spectrum of compound **4d**; Figure S10: ^13^C-NMR spectrum of compound **4e**; Figure S11: UV spectrum of compound **4a**; Figure S12: UV spectrum of compound **4b**; Figure S13: UV spectrum of compound **4c**; Figure S14. UV spectrum of compound **4d**; Figure S15: UV spectrum of compound **4e**; Figure S16: ESI-HRMS of compound **4a**; Figure S17: Mass spectrum of **4a** in positive ion ESI-MS/MS; Figure S18: Proposed fragmentation of protonated **4a**; Figure S19: ESI-HRMS of compound **4b**; Figure S20: Mass spectrum of **4b** in positive ion ESI-MS/MS; Figure S21: Proposed fragmentation of protonated **4b**; Figure S22: ESI-HRMS of compound **4c**; Figure S23: Mass spectrum of **4c** in positive ion ESI-MS/MS; Figure S24: Proposed fragmentation of protonated **4c**; Figure S25: ESI-HRMS of compound **4d**; Figure S26: Mass spectrum of **4d** in positive ion ESI-MS/MS; Figure S27: Proposed fragmentation of protonated **4d**; Figure S28: ESI-HRMS of compound **4e**; Figure S29: Mass spectrum of **4e** in positive ion ESI-MS/MS; Figure S30: Proposed fragmentation of protonated **4e**.
