# Supplementary material for: Novel Flurbiprofen Derivatives as Antioxidant and Anti-Inflammatory Agents: Synthesis, In Silico, and In Vitro Biological Evaluation
Source: Molecules. 2024 Jan 12;29(2):385. doi: 10.3390/molecules29020385 (PMC10818523; doi:10.3390/molecules29020385)
Supplement: Supplementary file 1 [file molecules-29-00385-s001.zip › molecules-2773879-supplementary.pdf]

Supplementary Materials:

Novel Flurbiprofen Derivatives as Antioxidant and Anti-inflammatory Agents: Synthesis, In silico, and In vitro Biological Evaluation

Iliyan Ivanov[1*], Stanimir Manolov[1*], Dimitar Bojilov[1], Gabriel Marc[2], Diyana Dimitrova[1], Smaranda Oniga[3], Ovidiu Oniga[2], Paraskev Nedialkov[4] and Maria Stoyanova[1]

Table of Contents:

Figure S1. ^{1}H-NMR spectrum of compound **4a** page **3**

Figure S2. ^{1}H-NMR spectrum of compound **4b** page **4**

Figure S3. ^{1}H-NMR spectrum of compound **4c** page **5**

Figure S4. ^{1}H-NMR spectrum of compound **4d** page **6**

Figure S5. ^{1}H-NMR spectrum of compound **4e** page **7**

Figure S6. ^{13}C-NMR spectrum of compound **4a** page **8**

Figure S7. ^{13}C-NMR spectrum of compound **4b** page **9**

Figure S8. ^{13}C-NMR spectrum of compound **4c** page **10**

Figure S9. ^{13}C-NMR spectrum of compound **4d** page **11**

Figure S10. ^{13}C-NMR spectrum of compound **4e** page **12**

Figure S11. UV spectrum of compound **4a** page **13**

Figure S12. UV spectrum of compound **4b** page **14**

Figure S13. UV spectrum of compound **4c** page **15**

Figure S14. UV spectrum of compound **4d** page **16**

Figure S15. UV spectrum of compound **4e** page **17**

Figure S16. ESI-HRMS of compound **4a** page **18**

Figure S17. Mass spectrum of **4a** in positive ion ESI-MS/MS page **19**

Figure S18. Proposed fragmentation of protonated **4a** page **20**

Figure S19. ESI-HRMS of compound **4b** page **21**

Figure S20. Mass spectrum of **4b** in positive ion ESI-MS/MS page **22**

Figure S21. Proposed fragmentation of protonated **4b** page **23**

Figure S22. ESI-HRMS of compound **4c** page **24**

Figure S23. Mass spectrum of **4c** in positive ion ESI-MS/MS page **25**

Figure S24. Proposed fragmentation of protonated **4c** page **26**

Figure S25. ESI-HRMS of compound **4d** page **27**

Figure S26. Mass spectrum of **4d** in positive ion ESI-MS/MS page **28**

Figure S27. Proposed fragmentation of protonated **4d** page **29**

Figure S28. ESI-HRMS of compound **4e** page **30**

Figure S29. Mass spectrum of **4e** in positive ion ESI-MS/MS page **31**

Figure S30. Proposed fragmentation of protonated **4e** page **32**

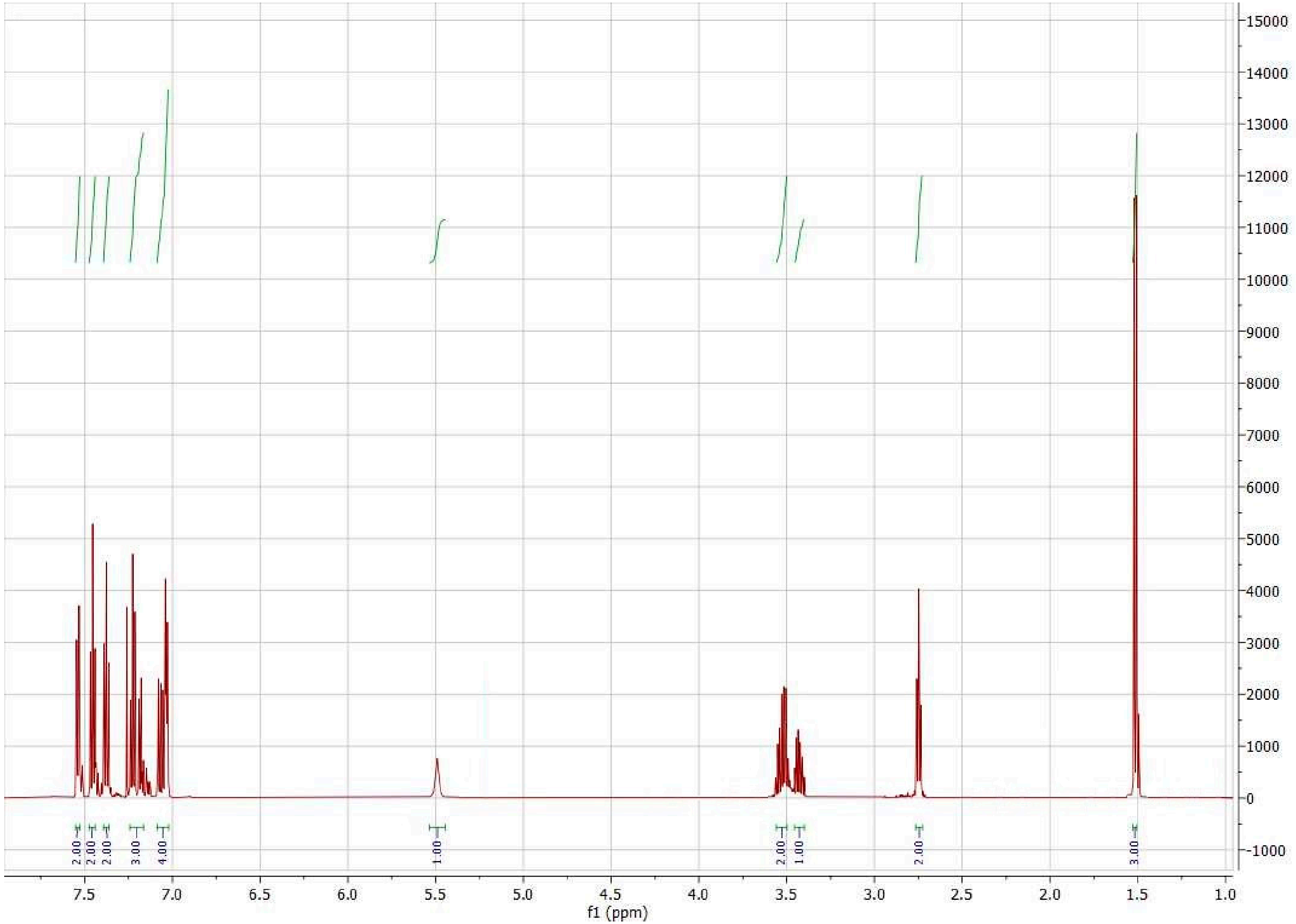

Figure S1. ^{1}H-NMR spectrum of compound **4a**.

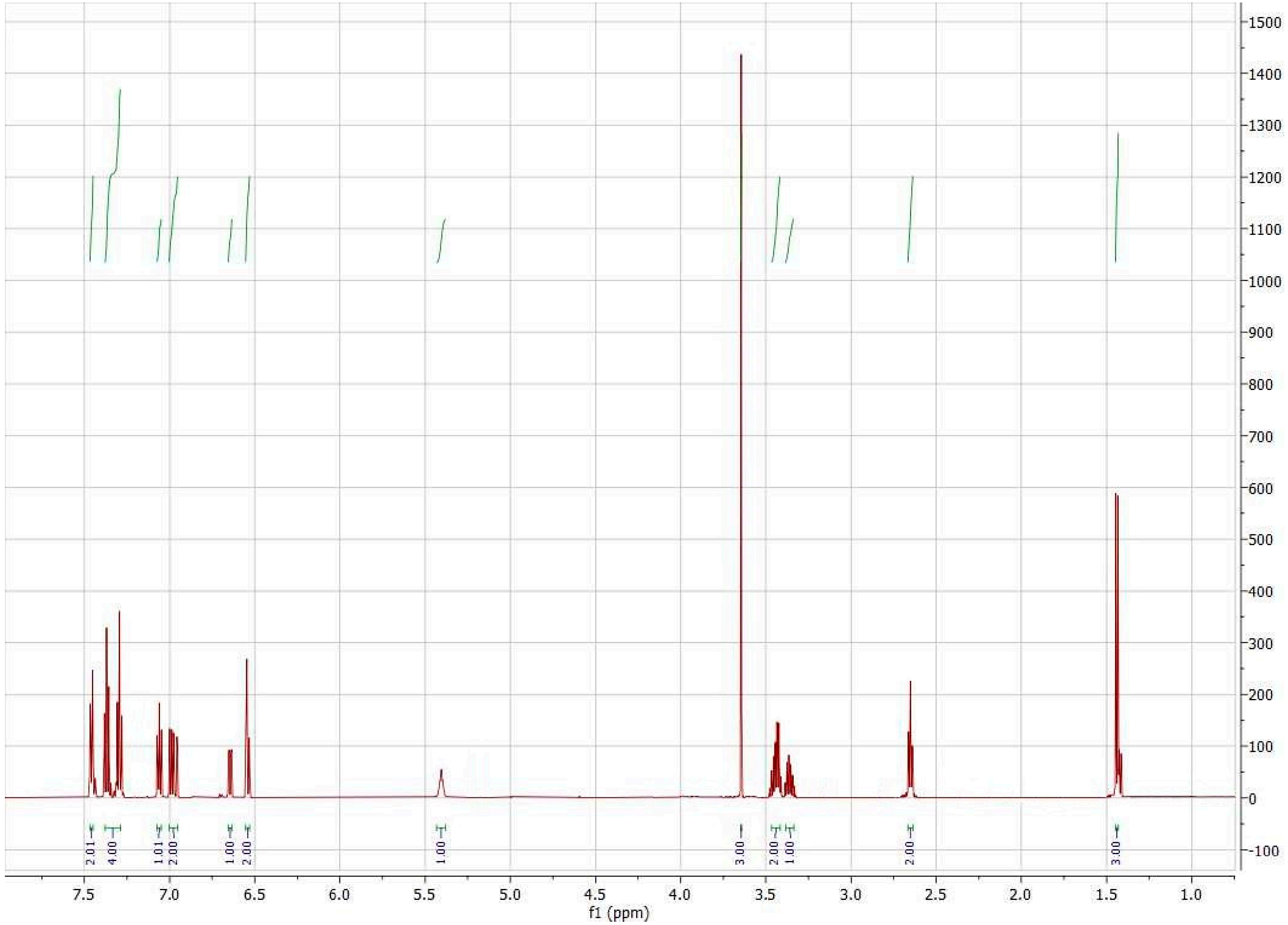

Figure S2. ^{1}H-NMR spectrum of compound **4b**.

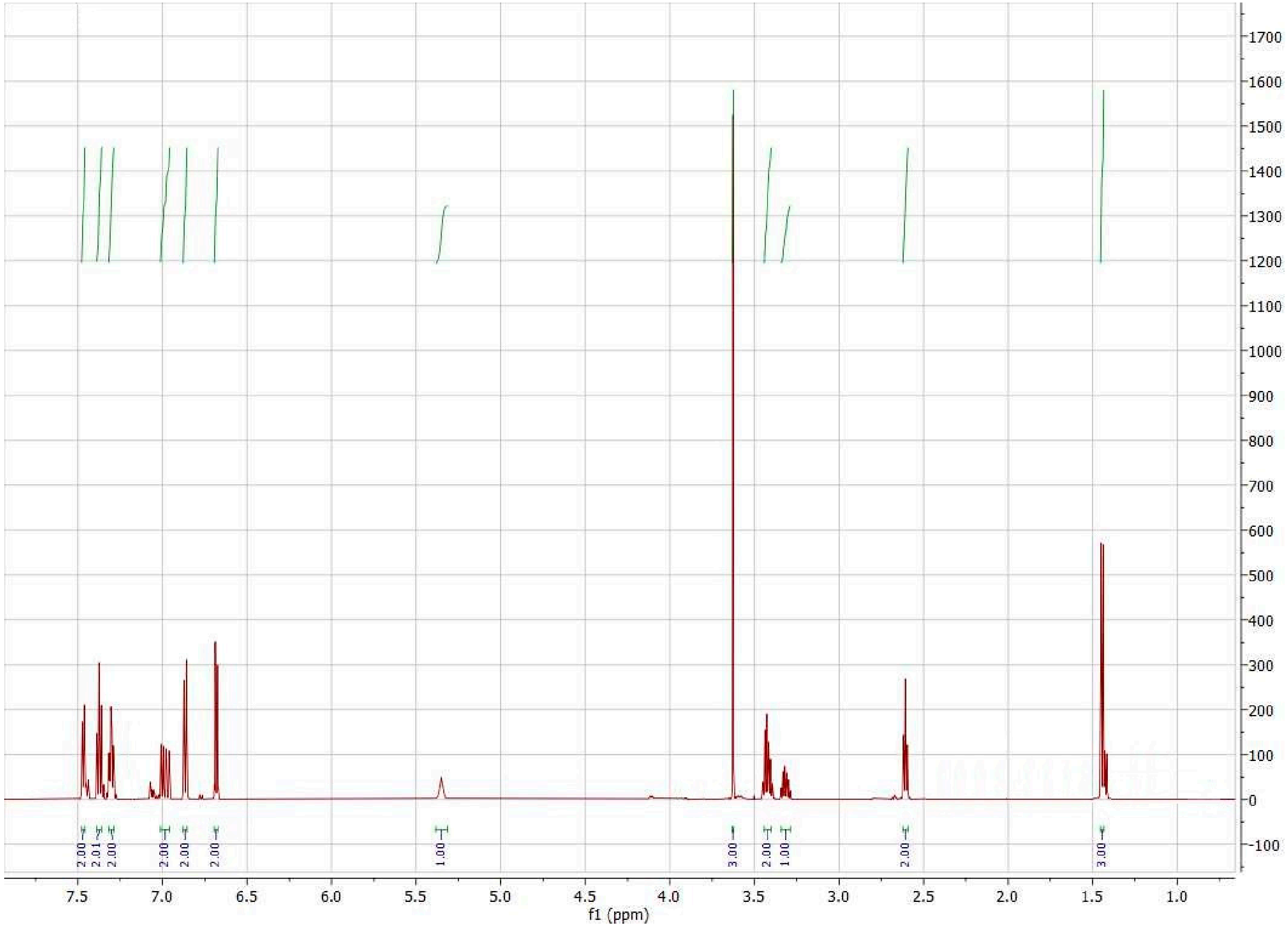

Figure S3. ^{1}H-NMR spectrum of compound **4c**.

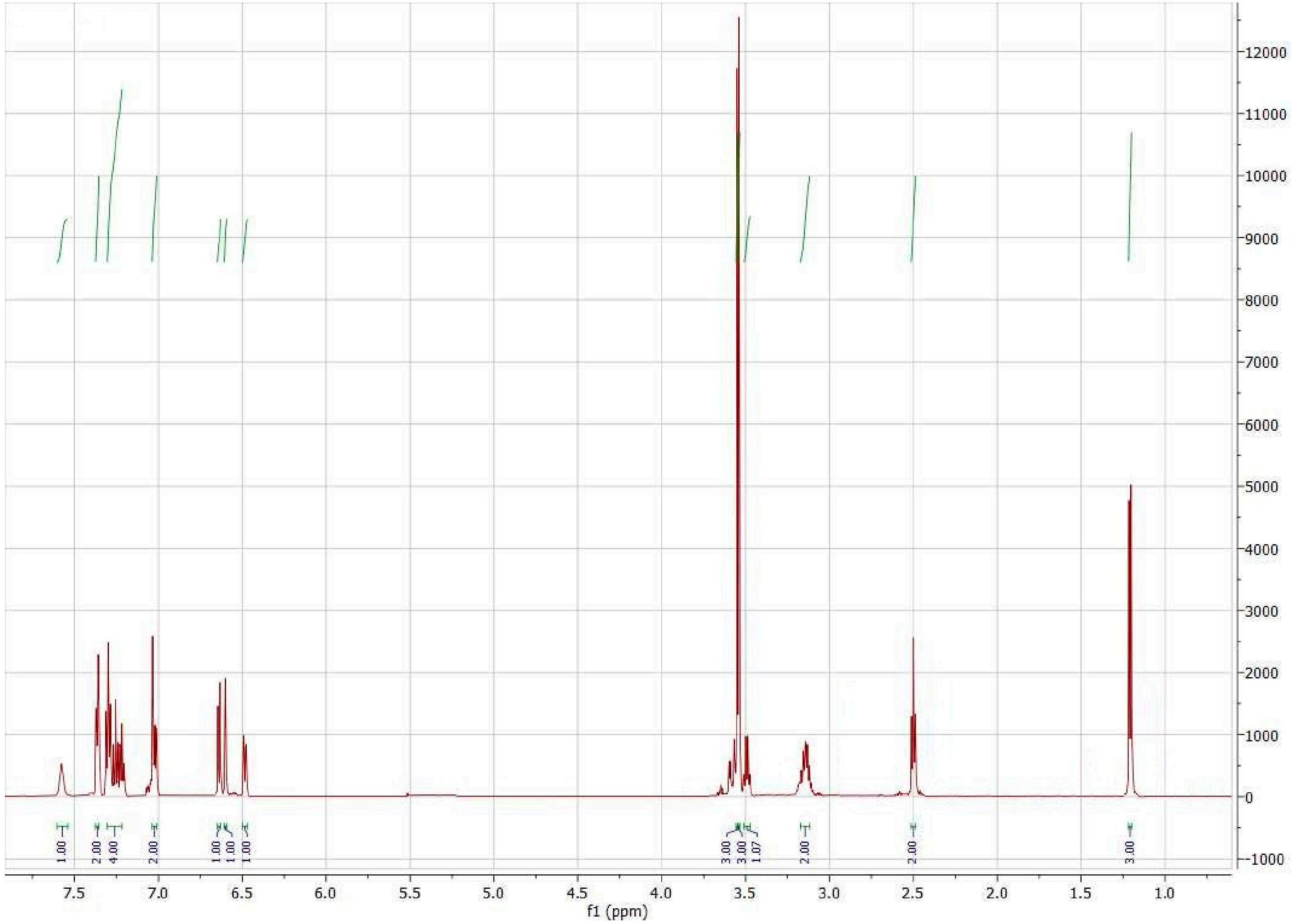

Figure S4. ^{1}H-NMR spectrum of compound **4d**.

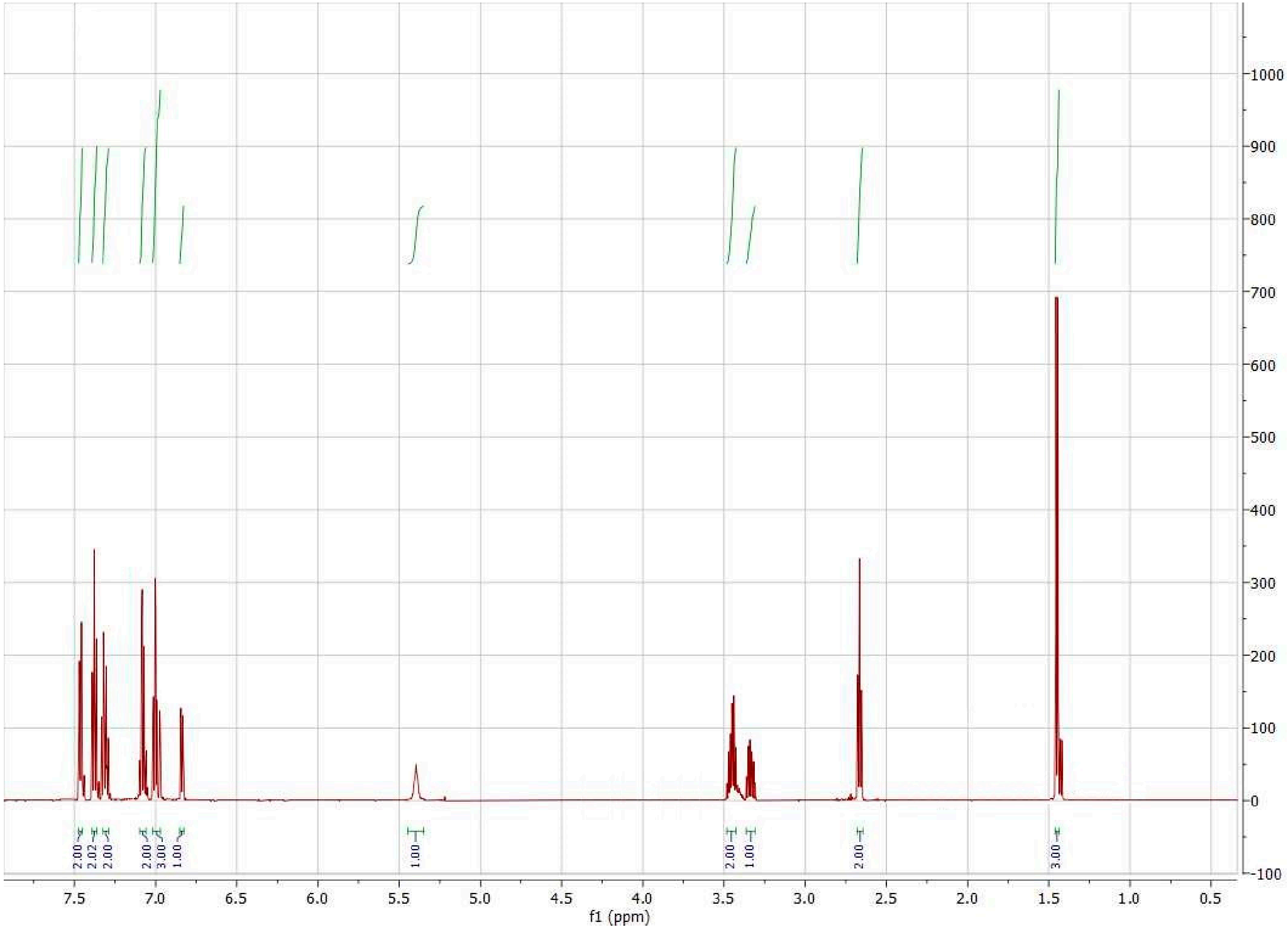

Figure S5. ^{1}H-NMR spectrum of compound **4e**.

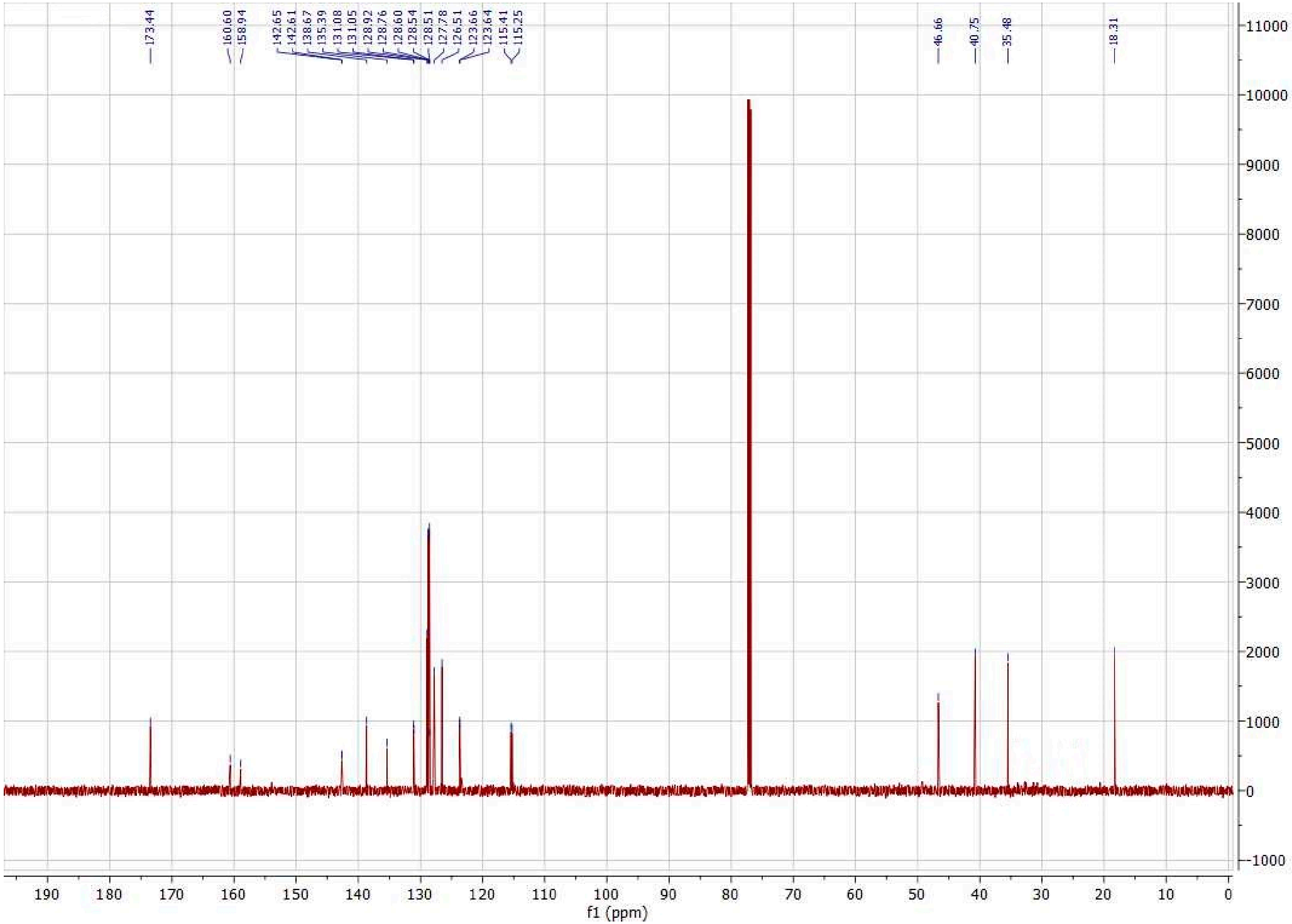

Figure S6. ^{13}C-NMR spectrum of compound **4a**.

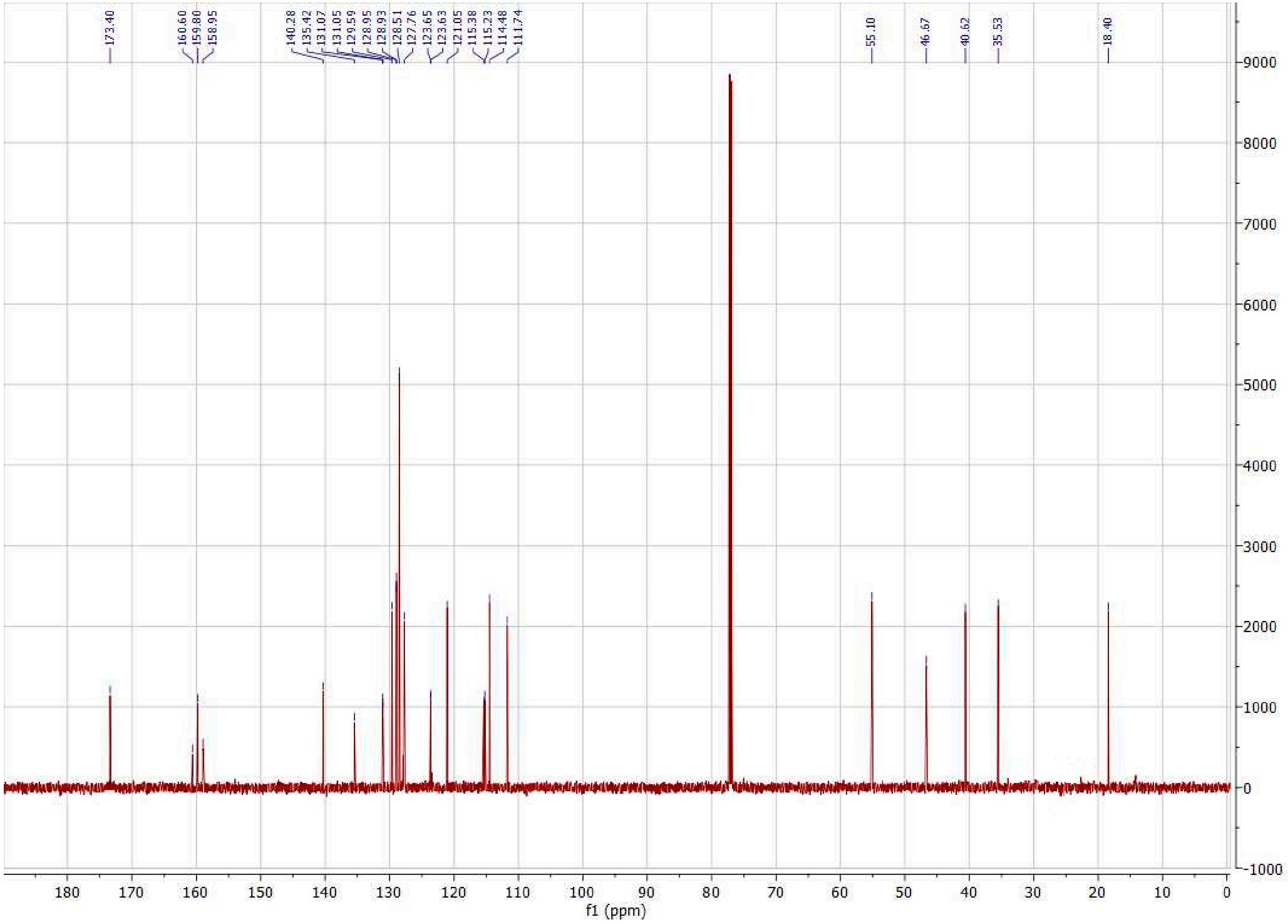

Figure S7. ^{13}C-NMR spectrum of compound **4b**.

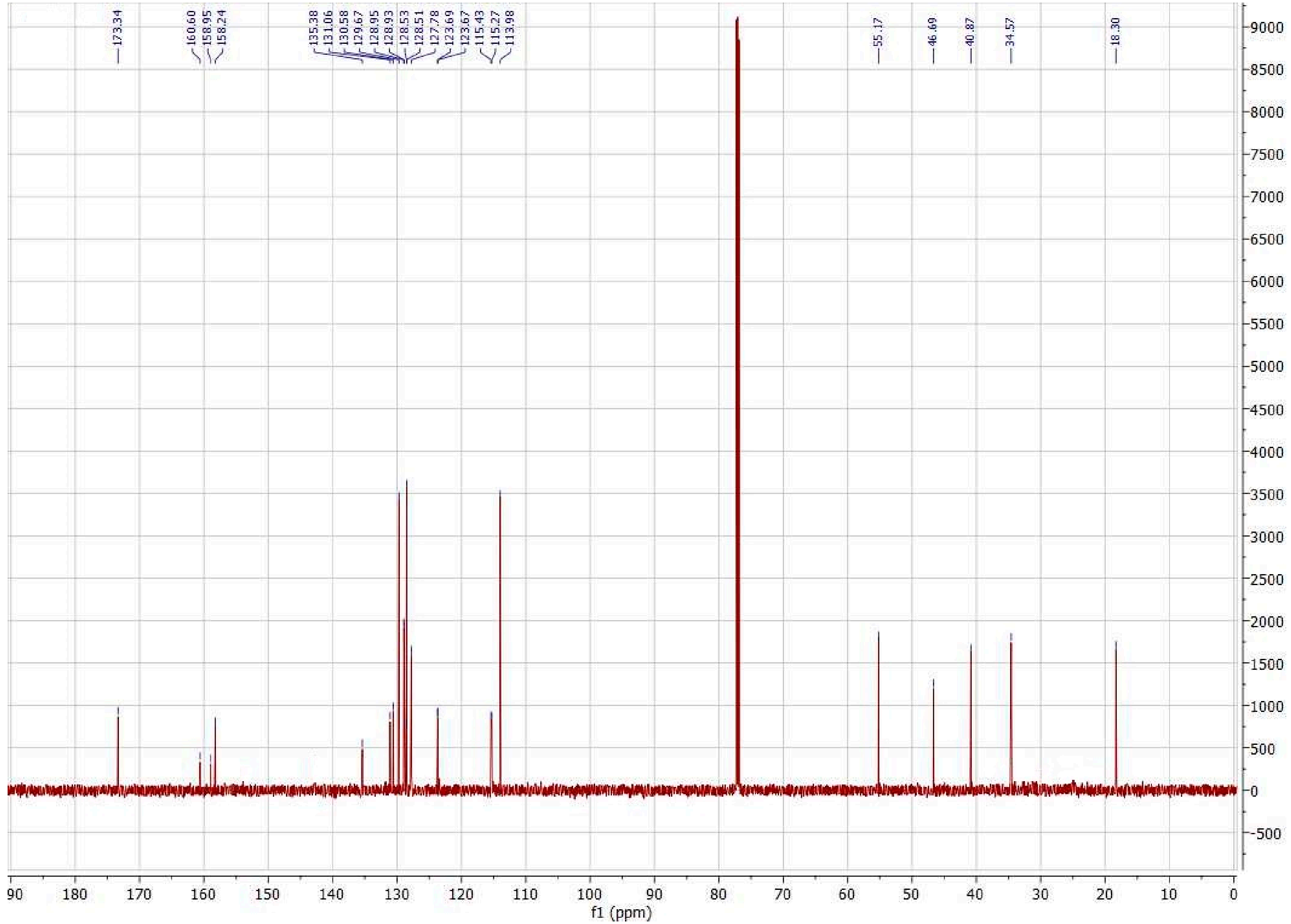

Figure S8. ^{13}C-NMR spectrum of compound **4c**.

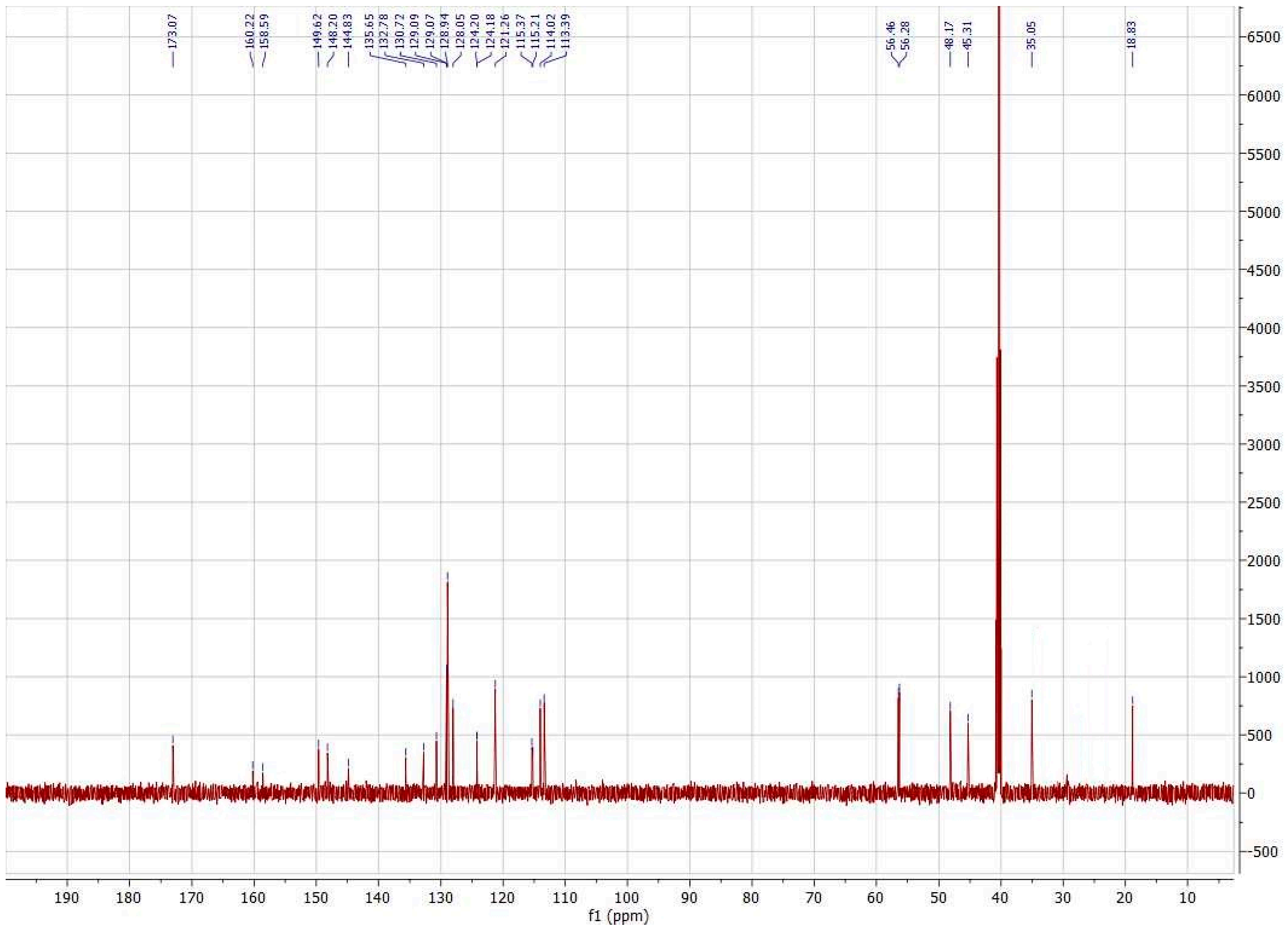

Figure S9. ^{13}C-NMR spectrum of compound **4d**.

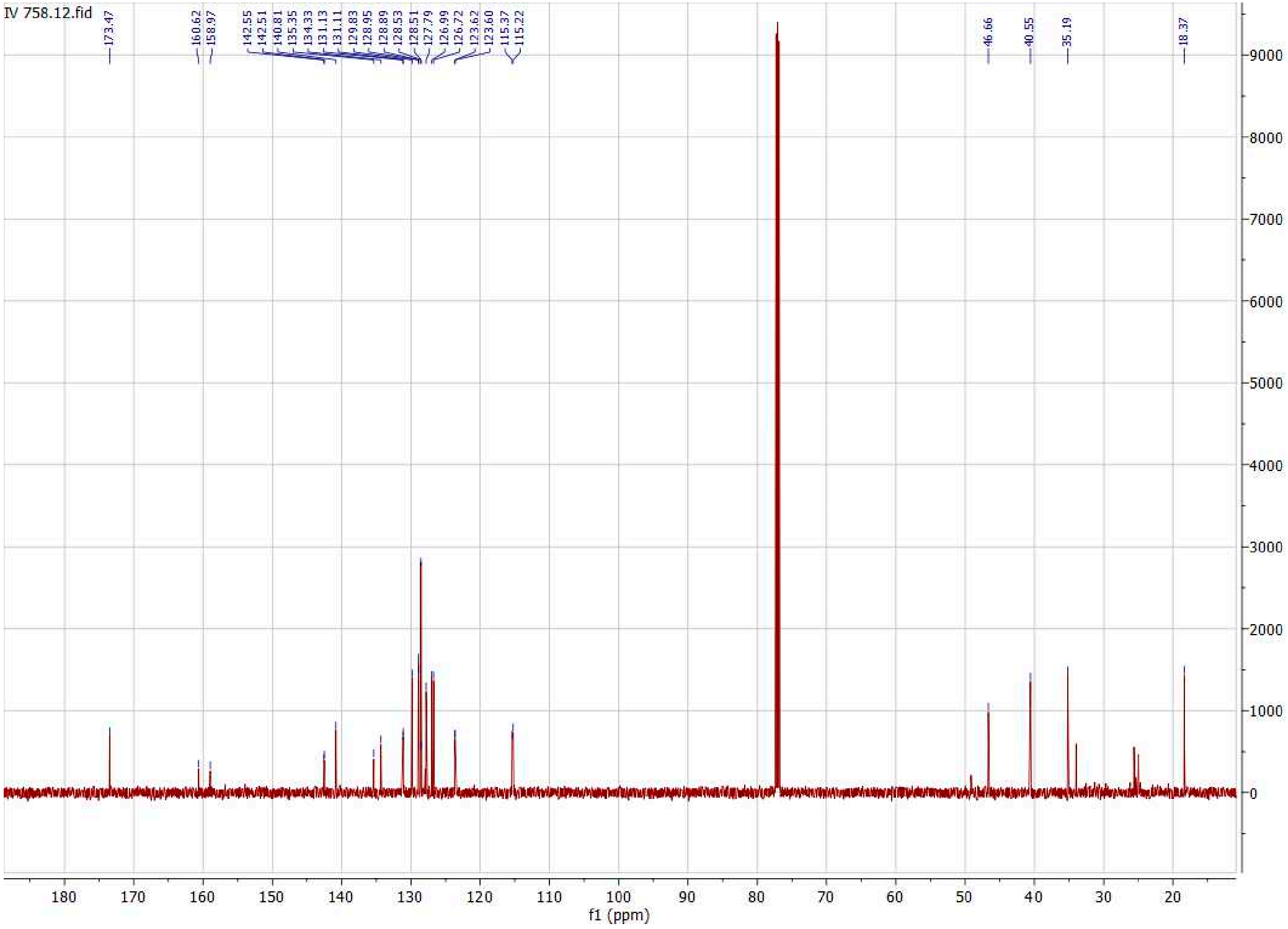

Figure S10. ^{13}C-NMR spectrum of compound **4e**.

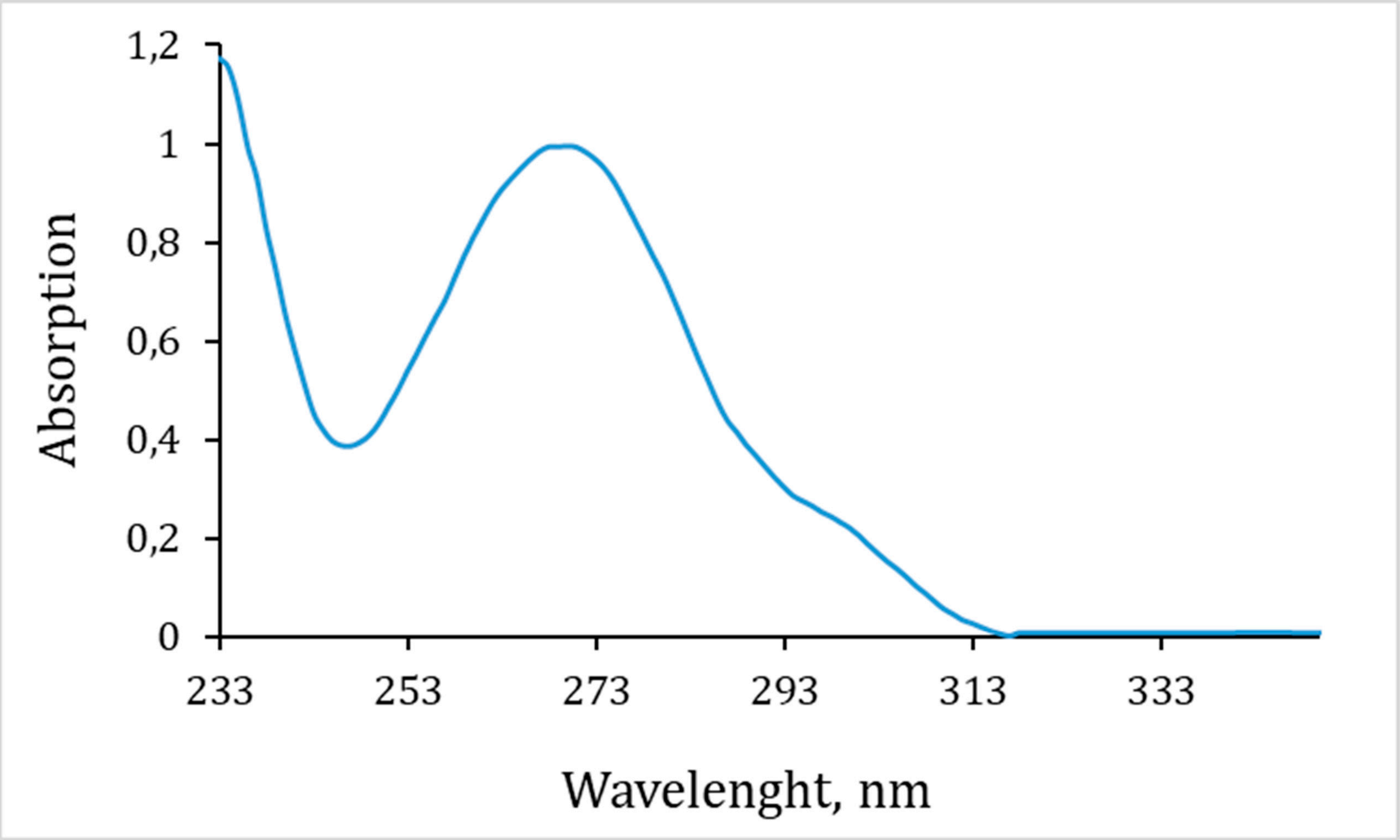

Figure S11. UV spectrum of compound **4a**.

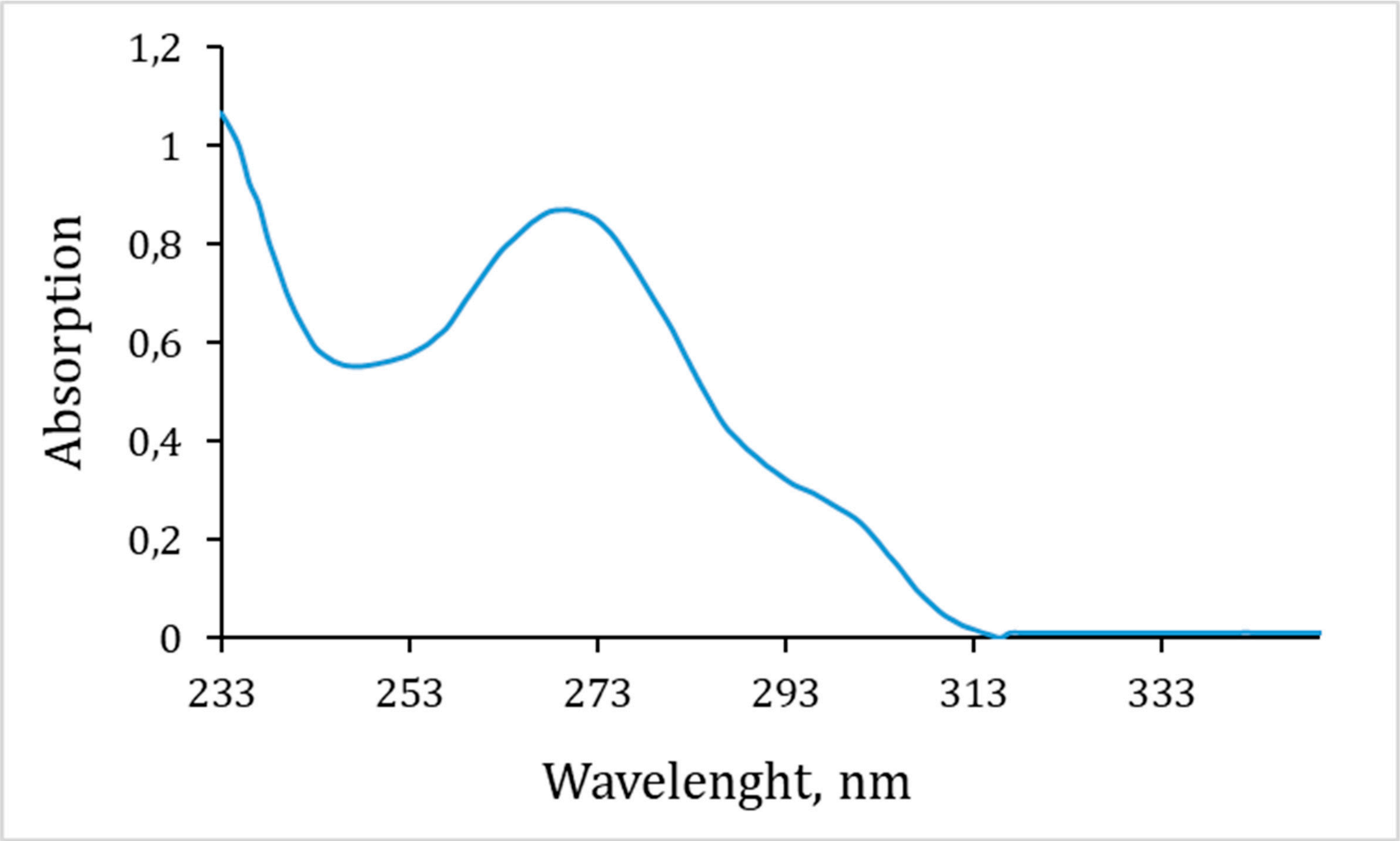

Figure S12. UV spectrum of compound **4b**.

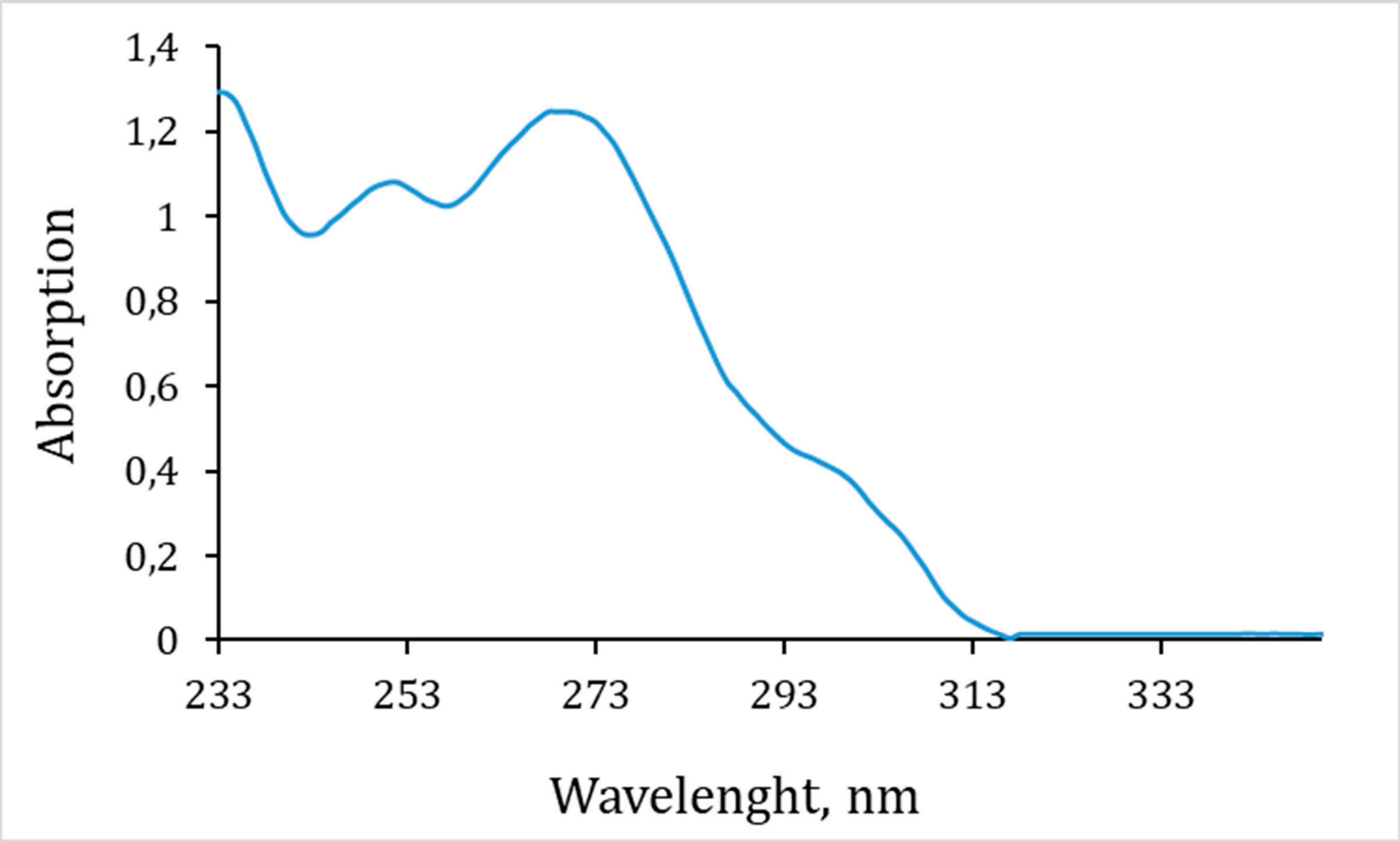

Figure S13. UV spectrum of compound **4c**.

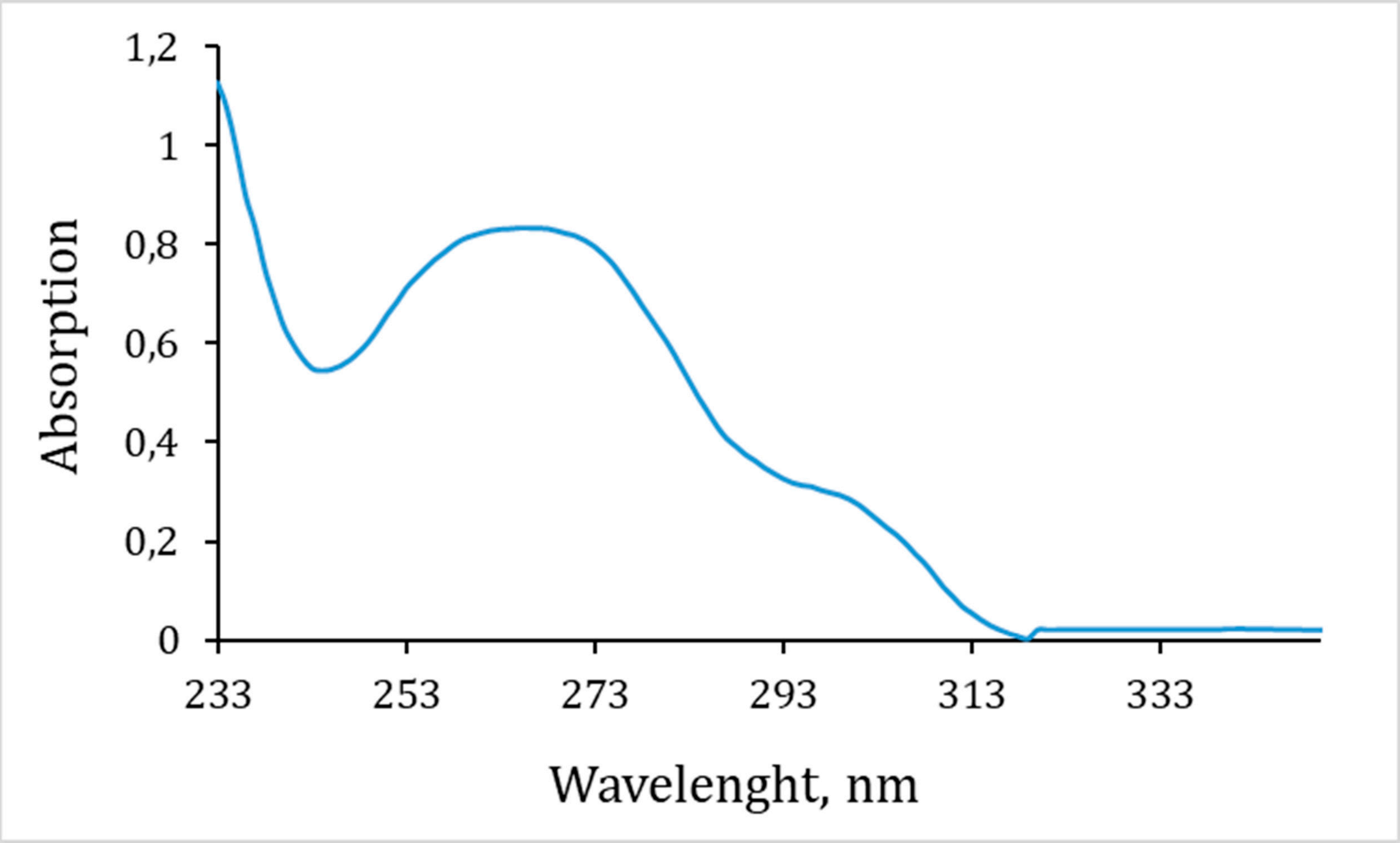

Figure S14. UV spectrum of compound **4d**.

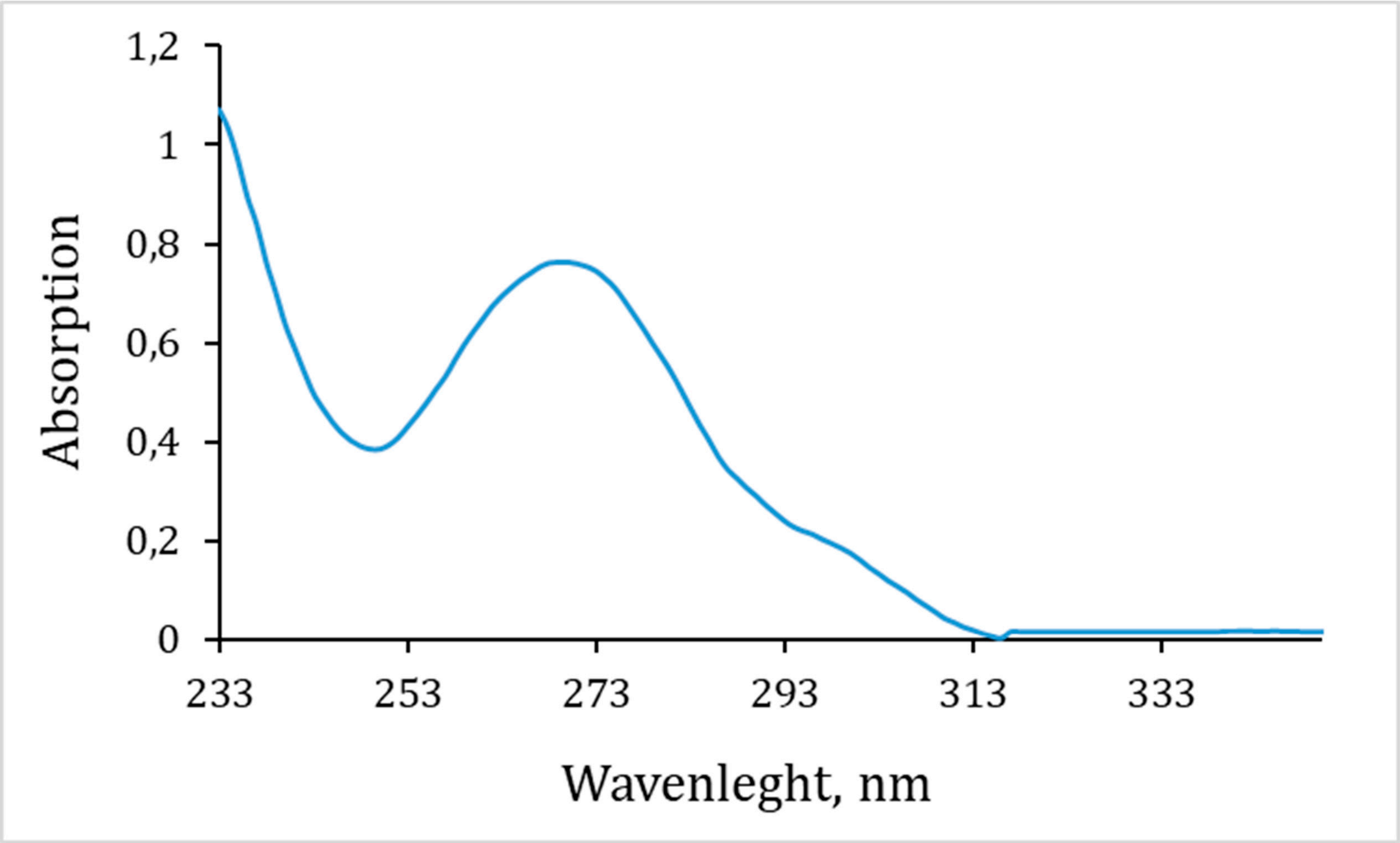

Figure S15. UV spectrum of compound **4e**.

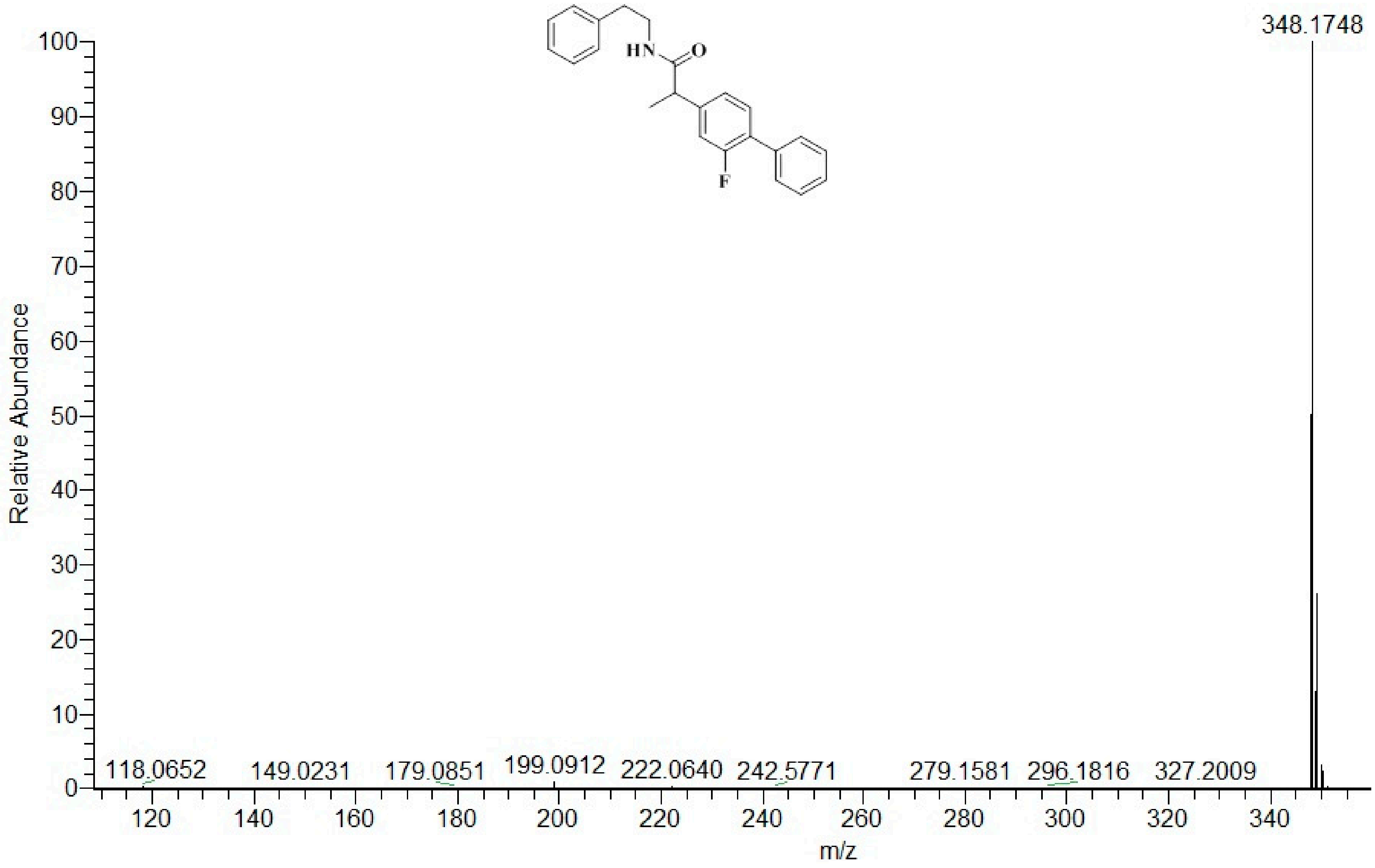

Figure S16. ESI-HRMS of compound **4a**.

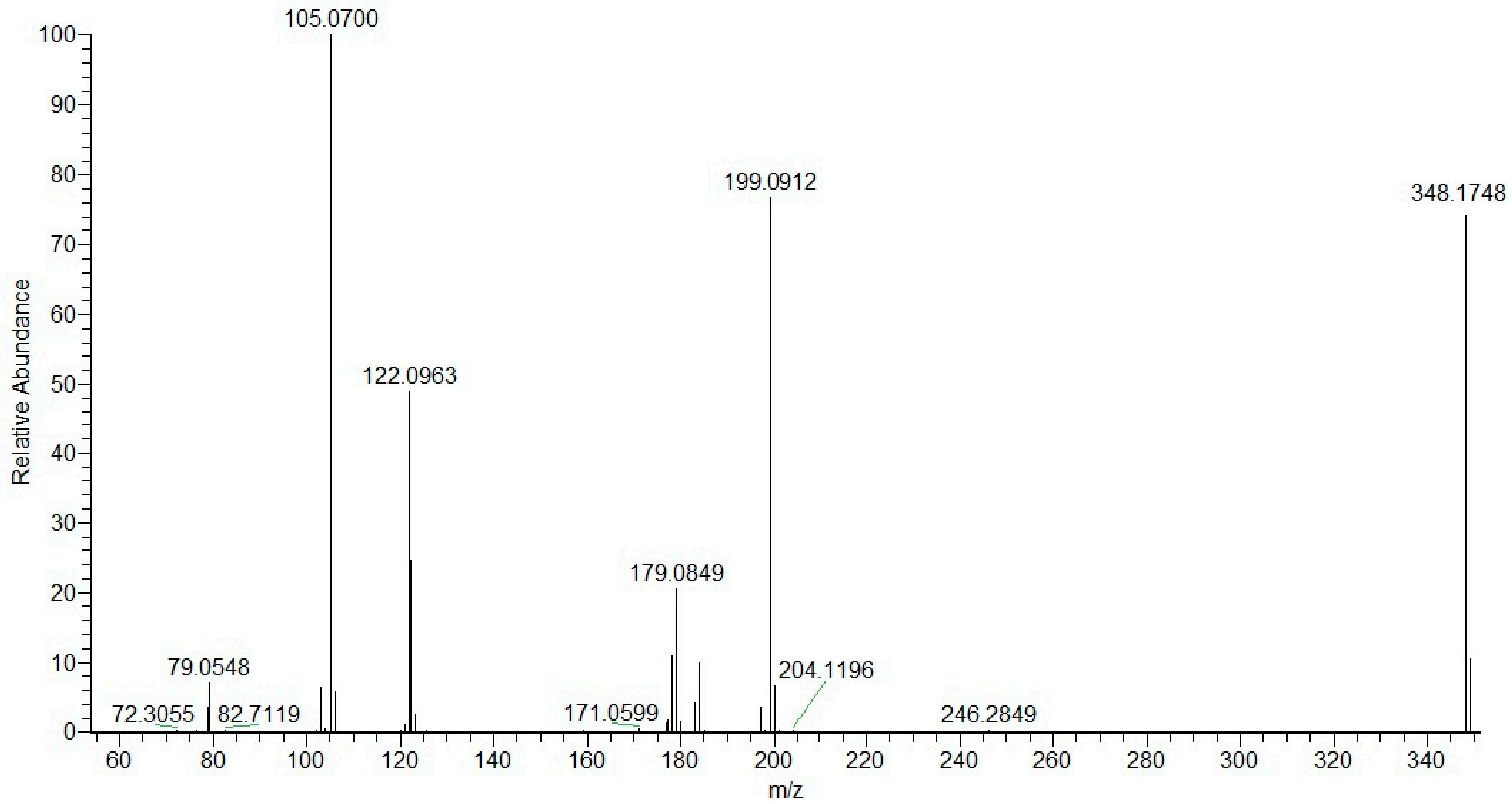

Figure S17. Mass spectrum of **4a** obtained by positive ion ESI-MS/MS.

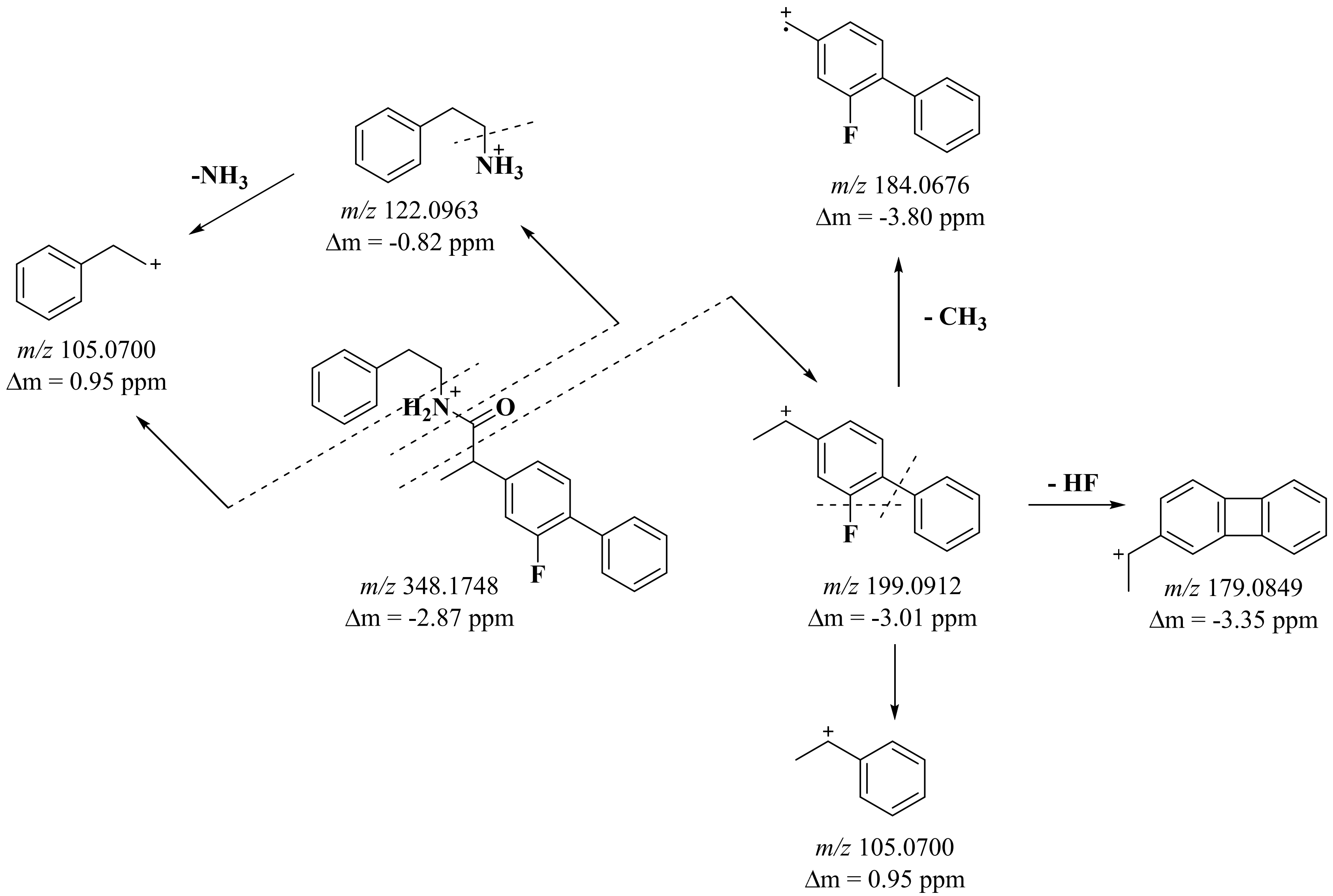

Figure S18. Proposed fragmentation of protonated **4a**.

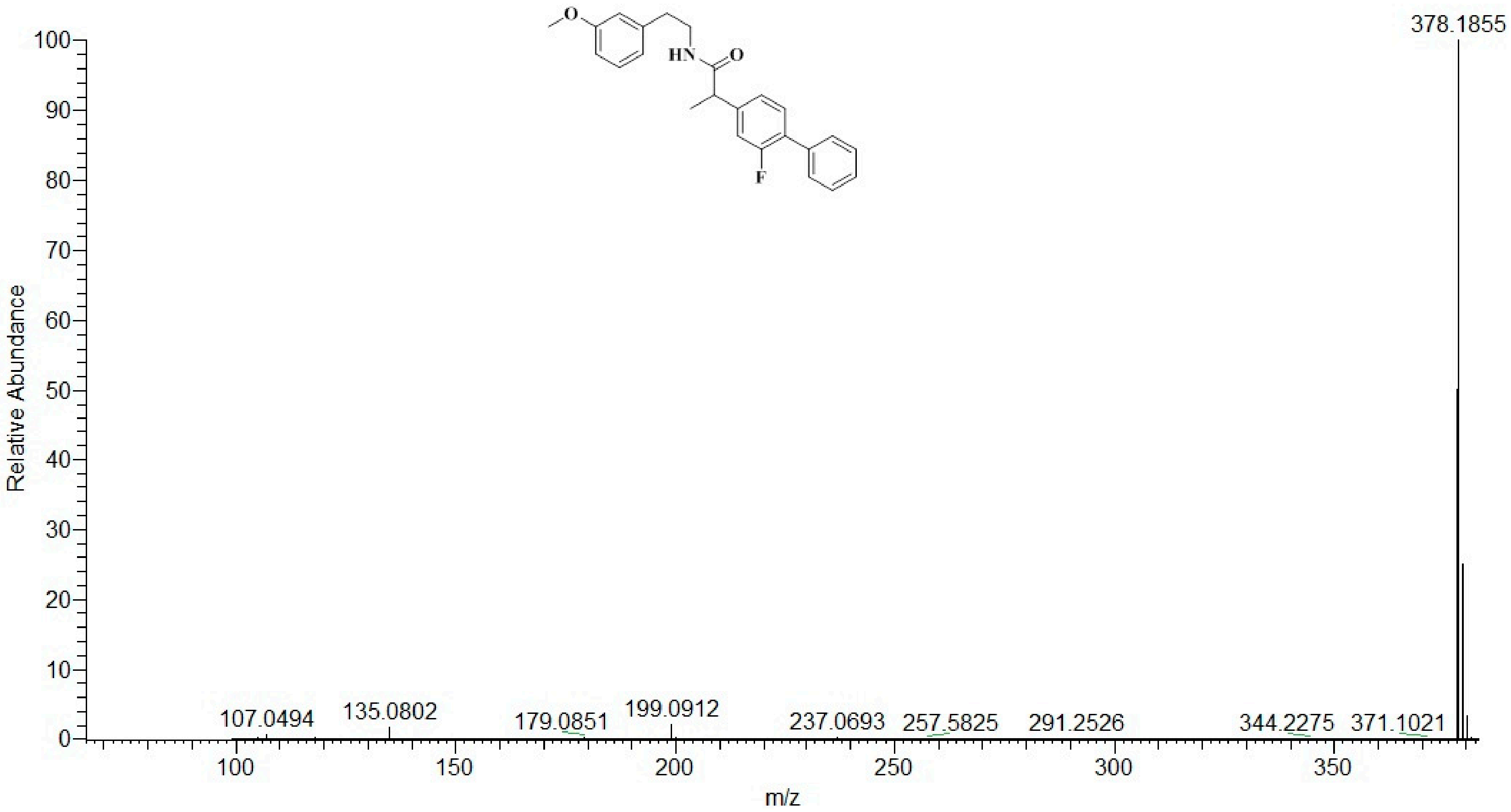

Figure S19. ESI-HRMS of compound **4b.**

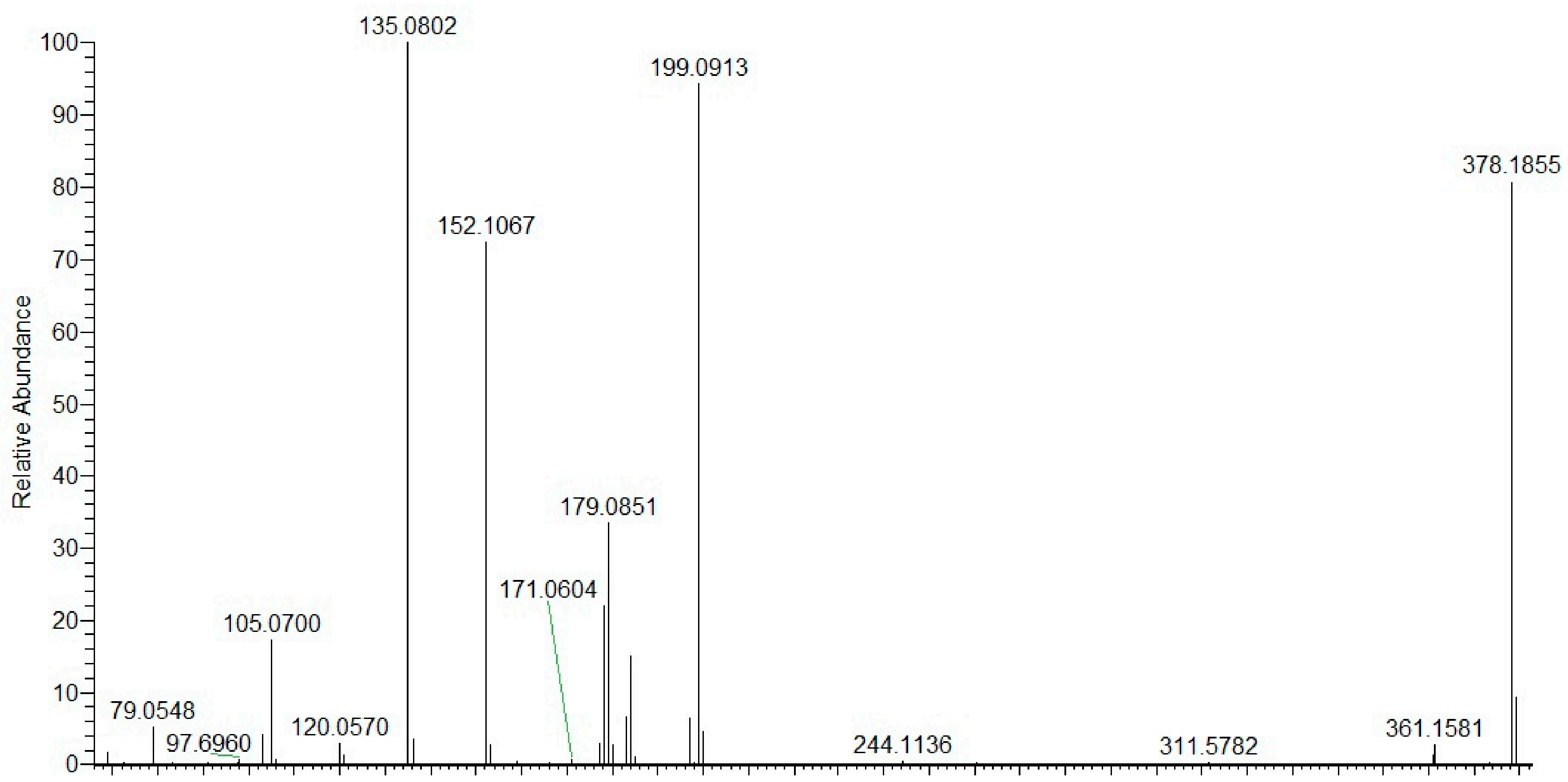

Figure S20. Mass spectrum of **4b** obtained by positive ion ESI-MS/MS.

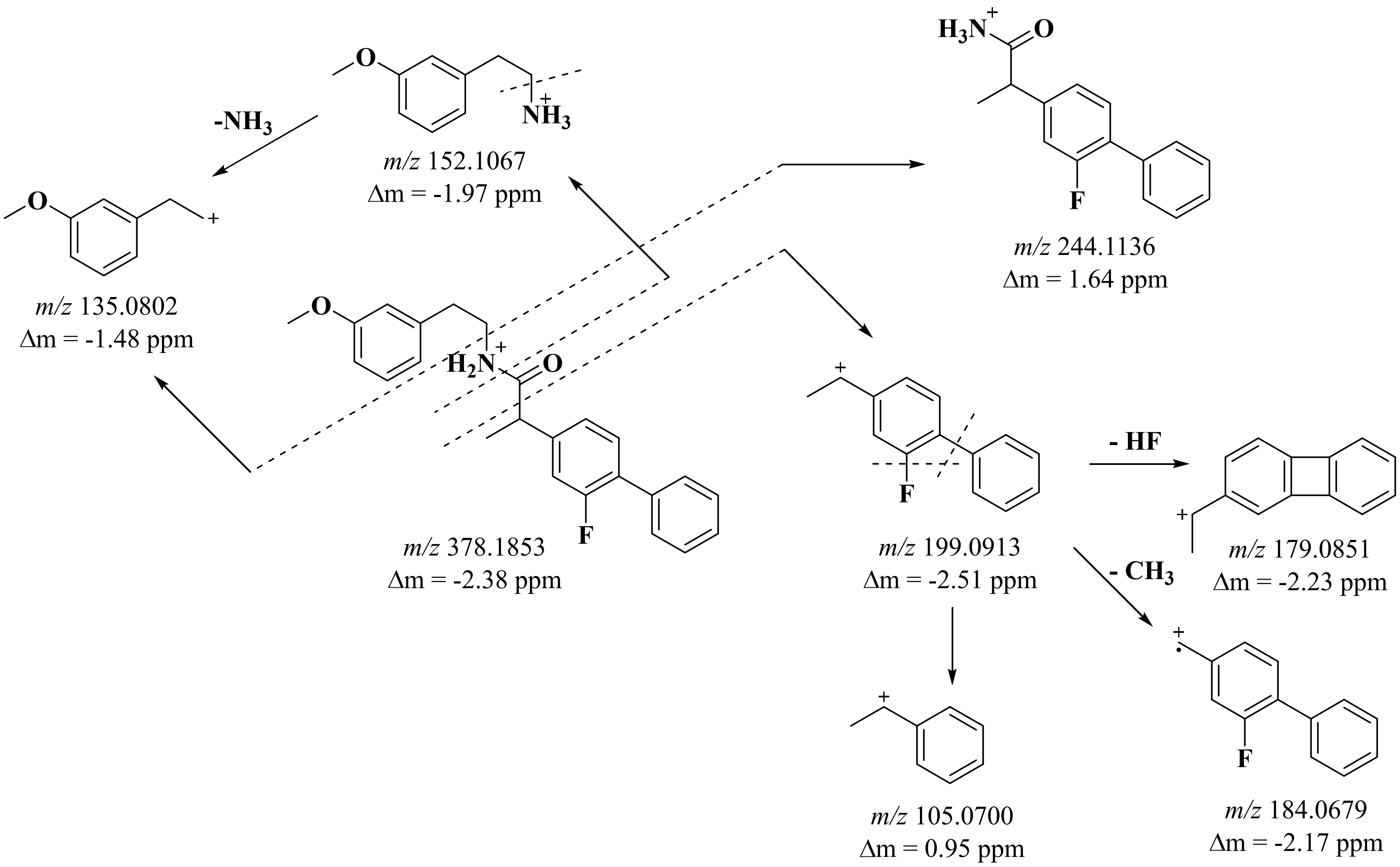

Figure S21. Proposed fragmentation of protonated **4b**.

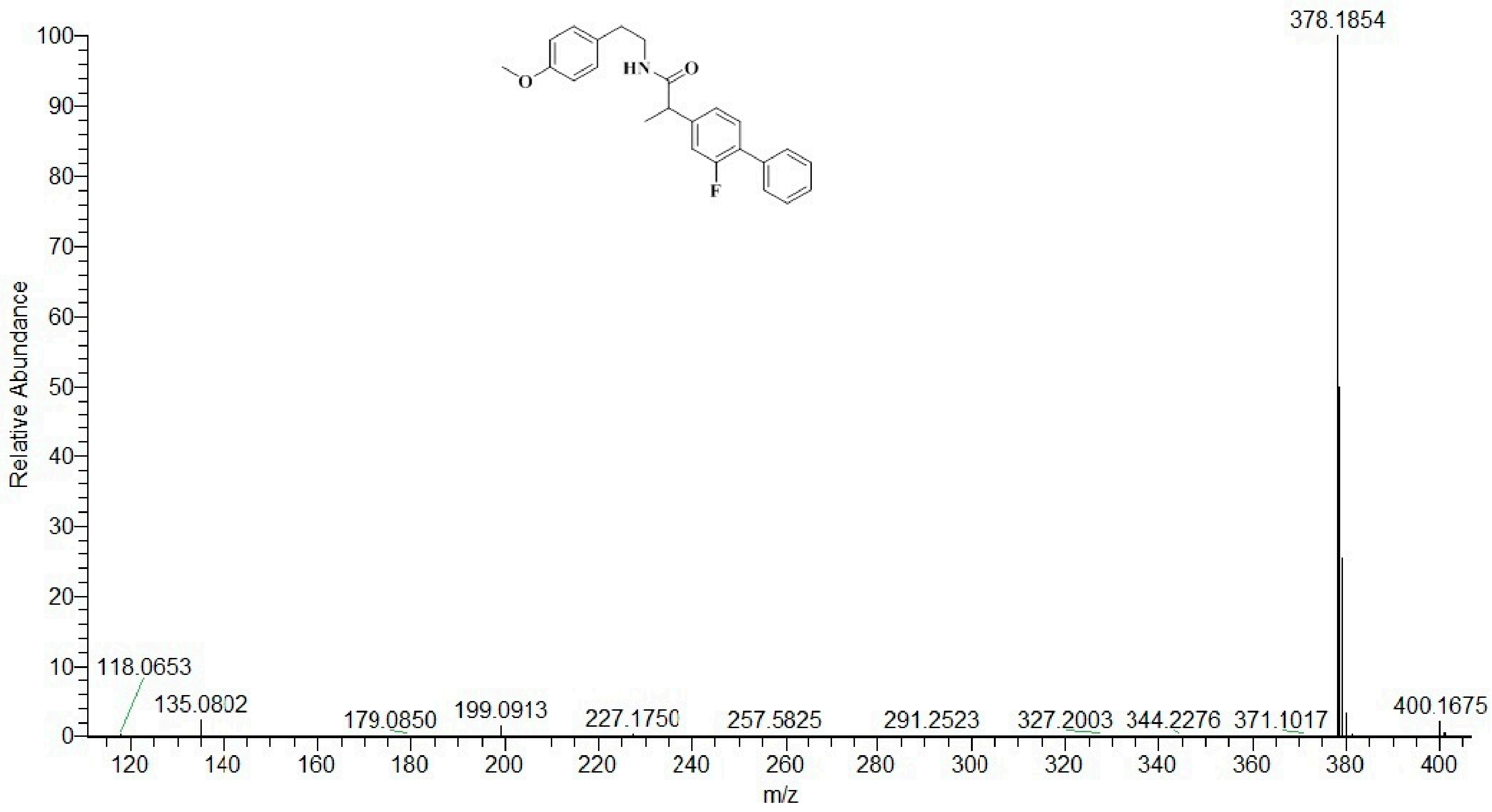

Figure S22. ESI-HRMS of compound **4c**.

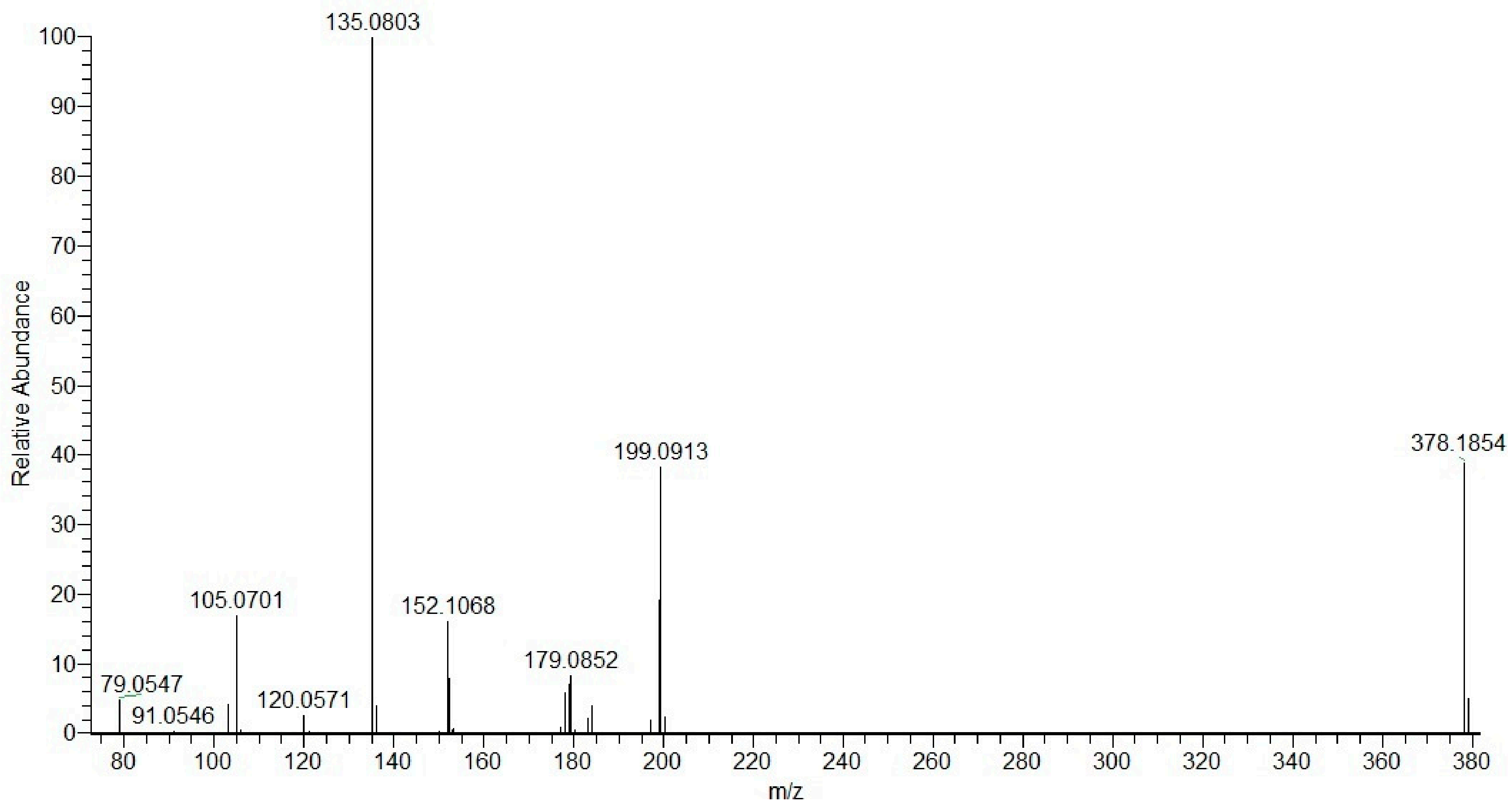

Figure S23. Mass spectrum of **4c** obtained by positive ion ESI-MS/MS**.**

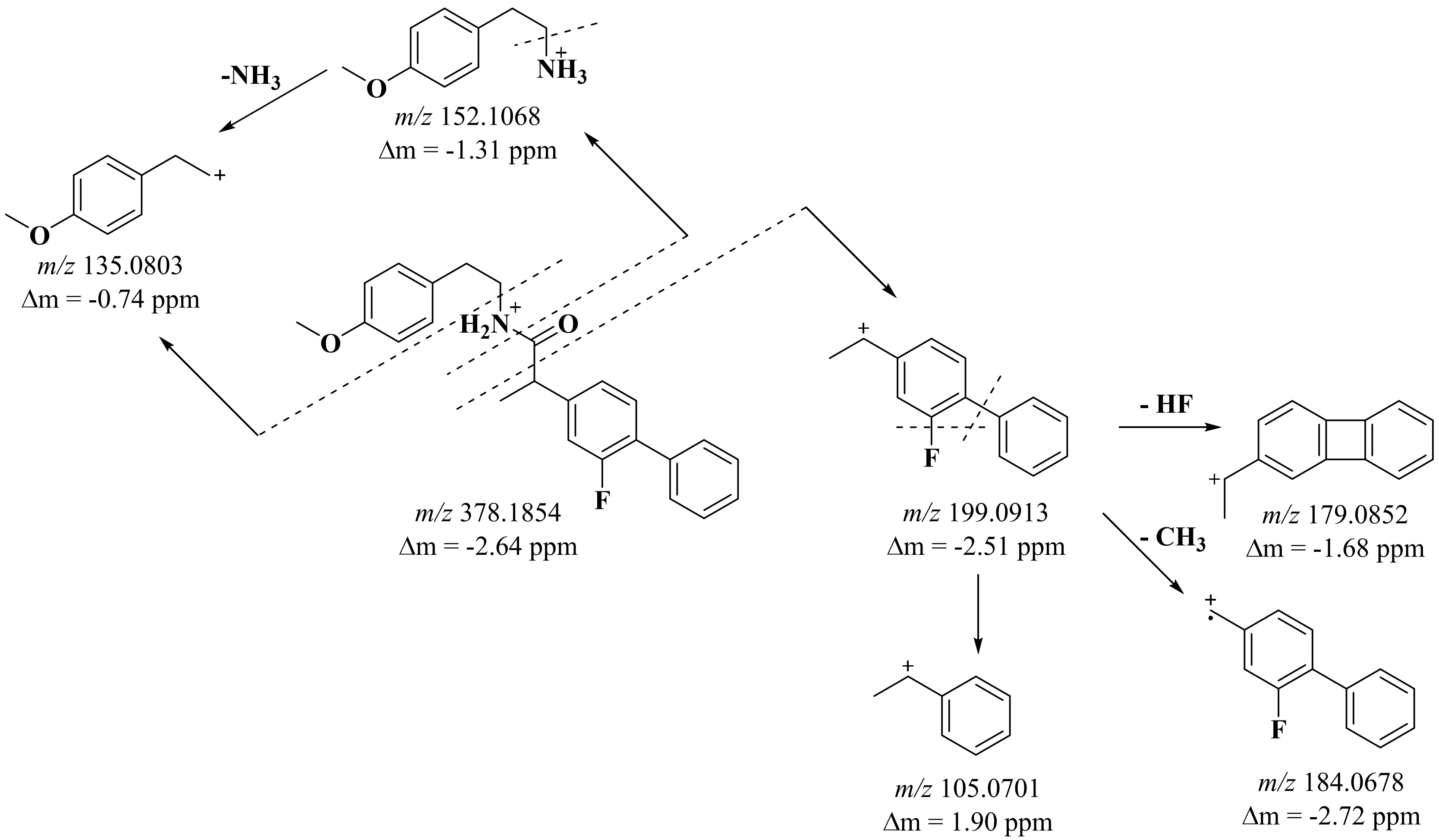

Figure S24. Proposed fragmentation of protonated **4c**.

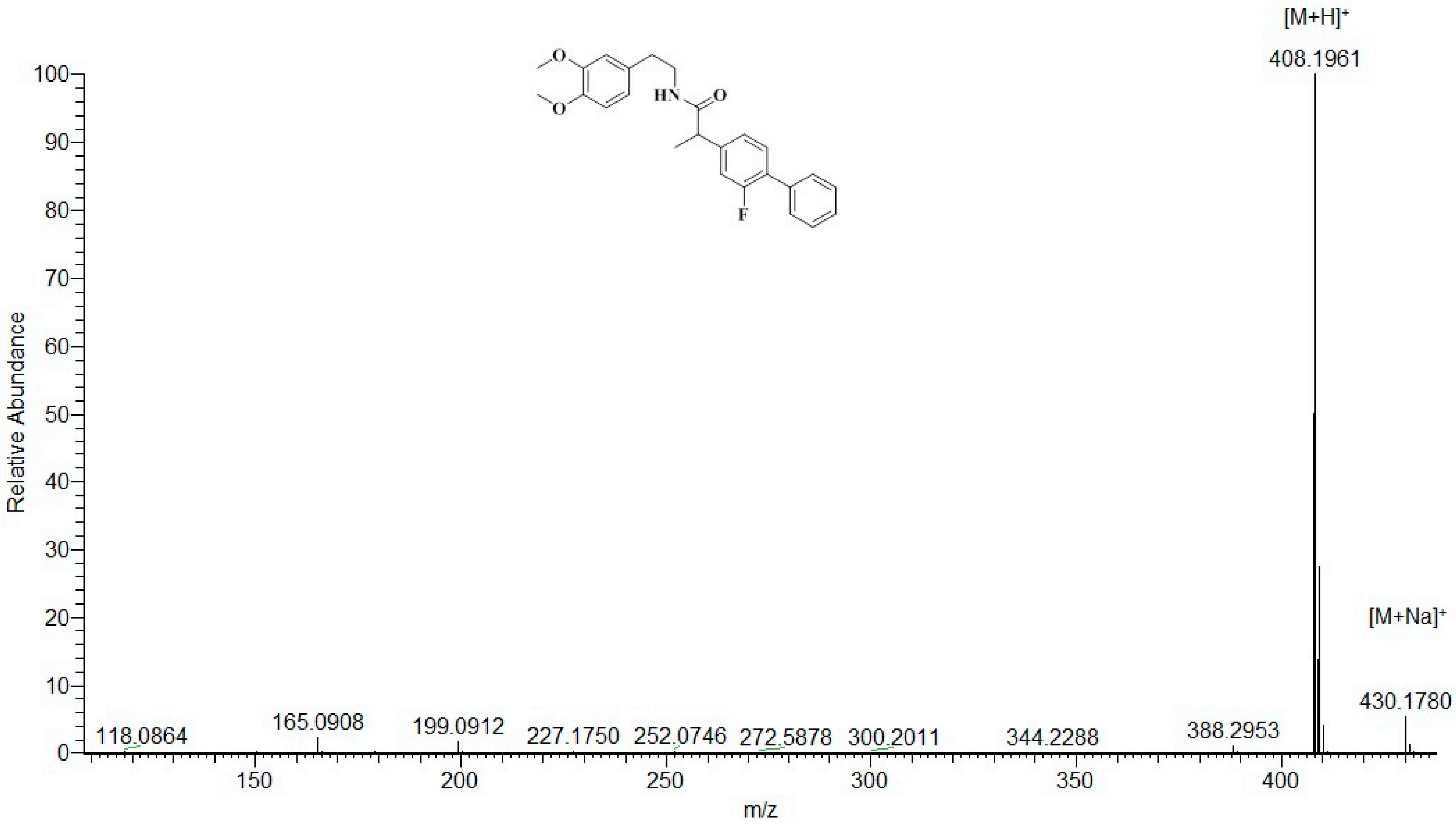

Figure S25. ESI-HRMS of compound **4d**.

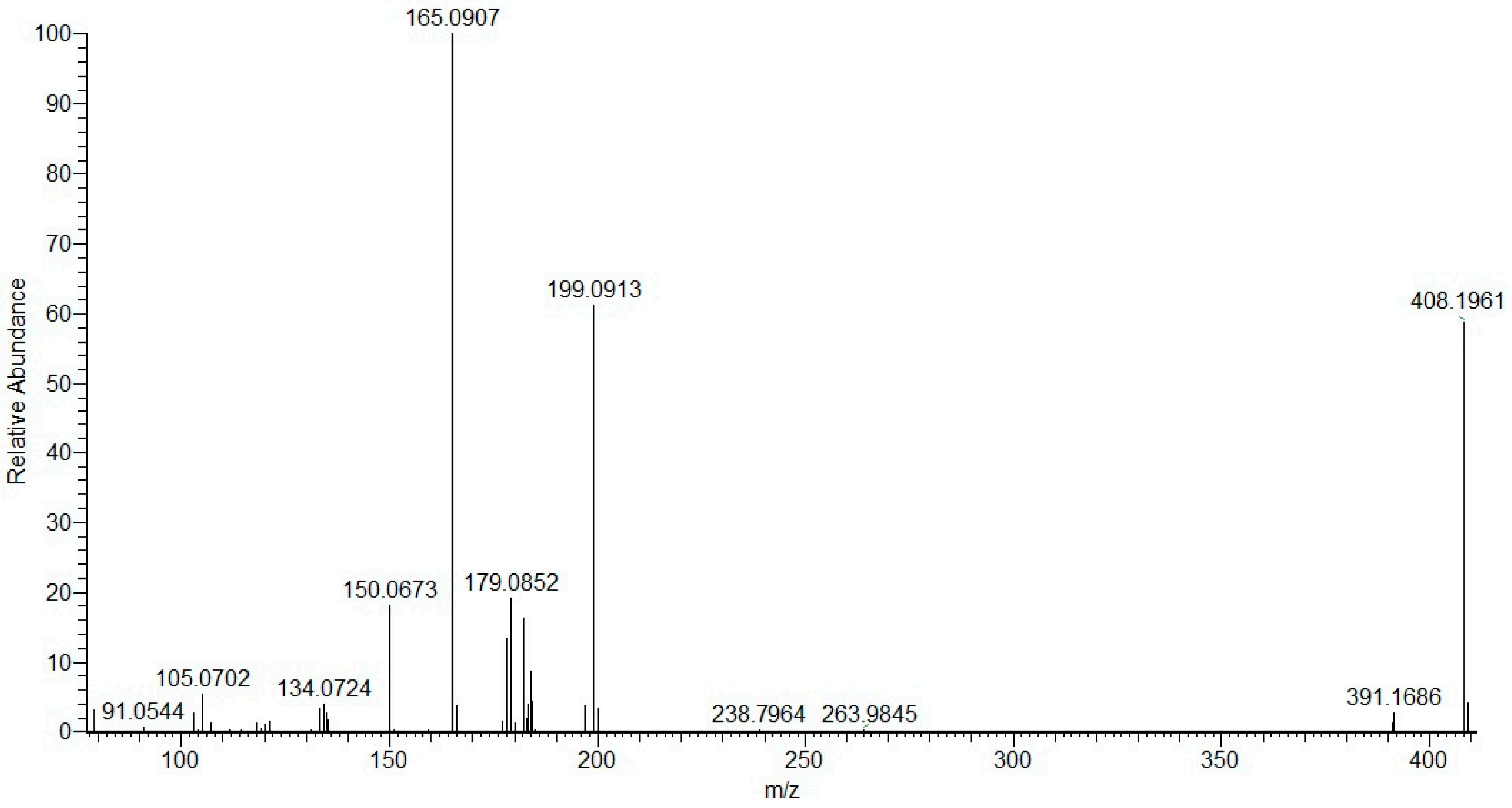

Figure S26. Mass spectrum of **4d** obtained by positive ion ESI-MS/MS**.**

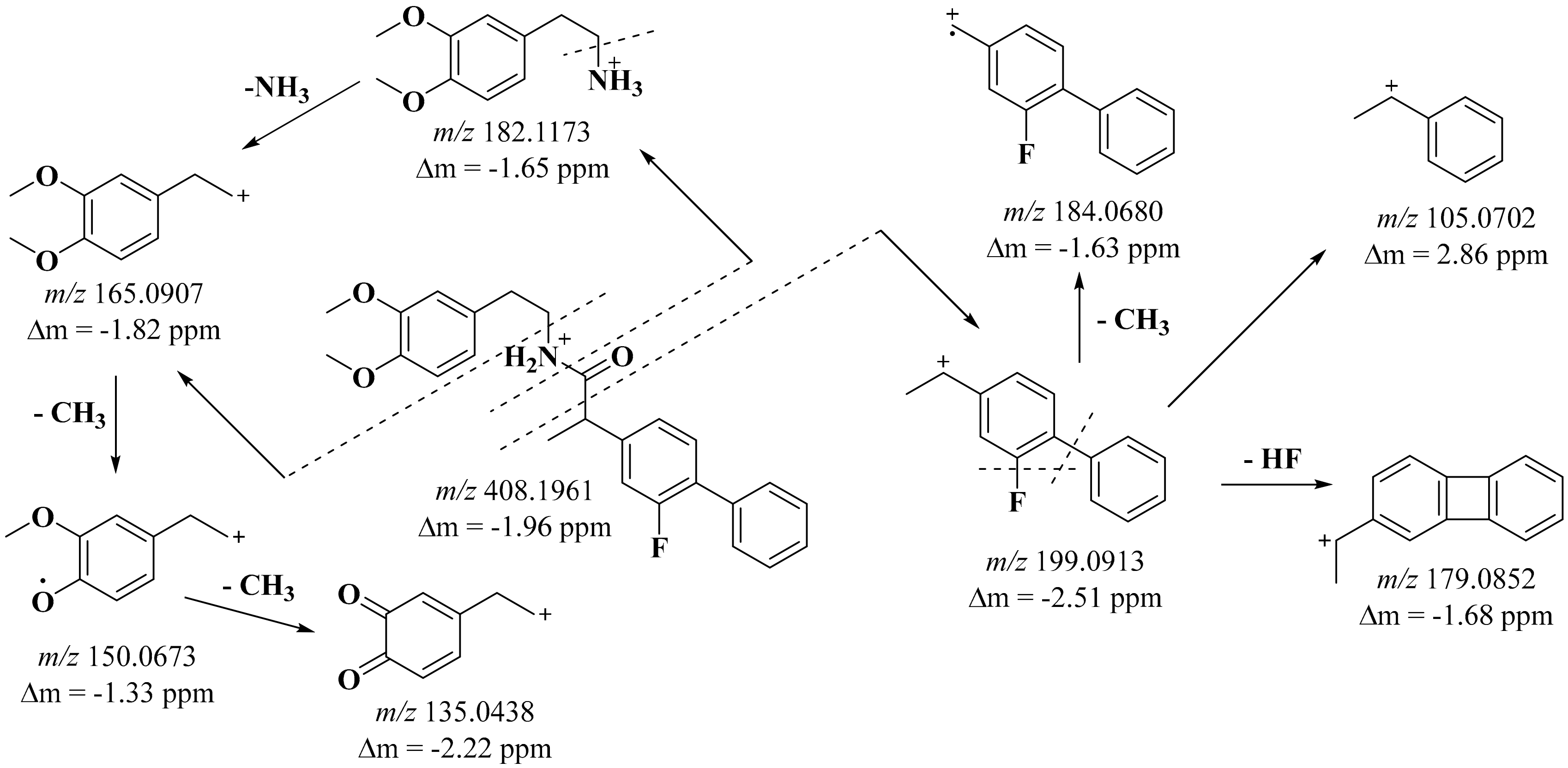

Figure S27. Proposed fragmentation of protonated **4d**.

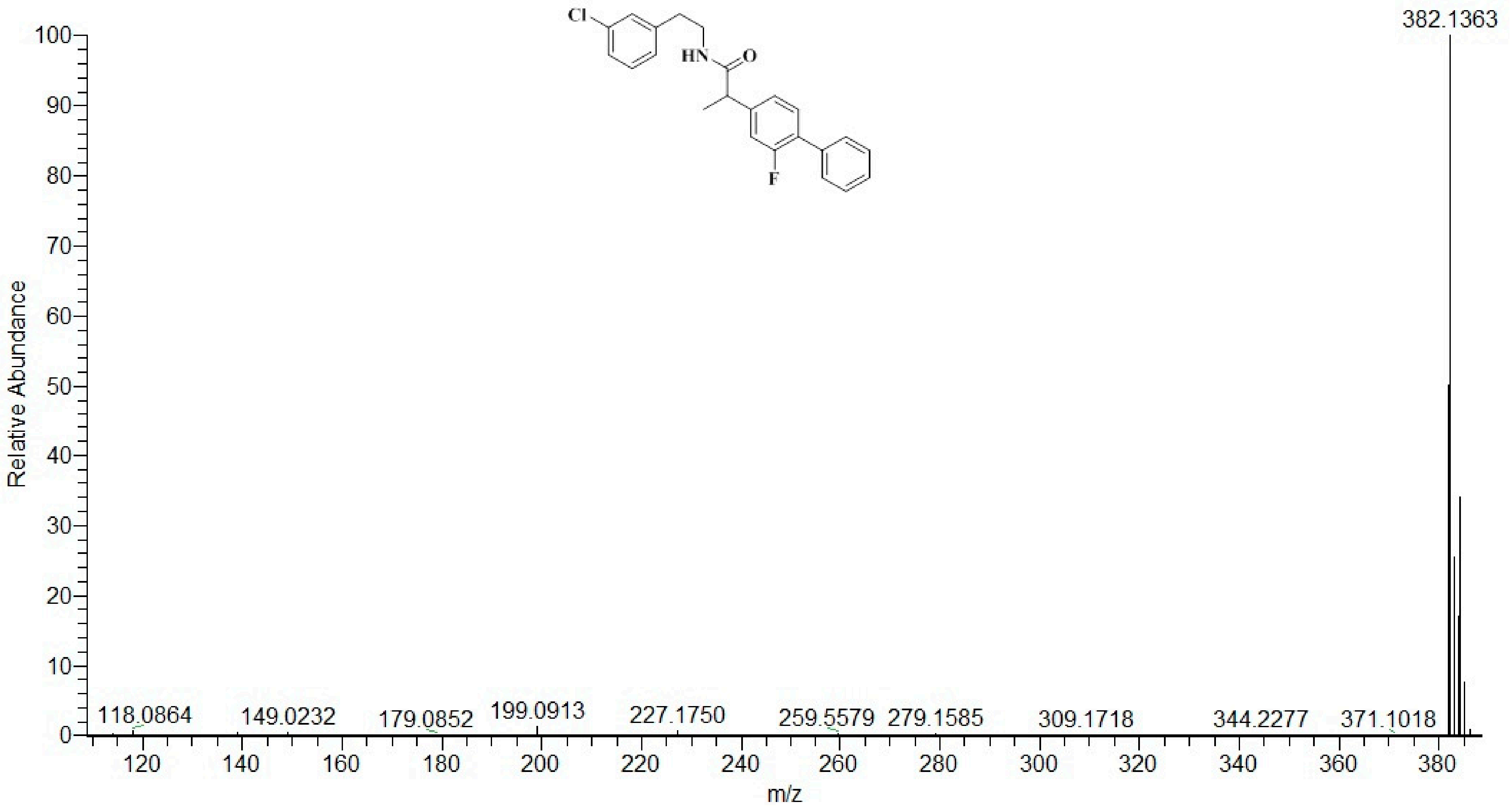

Figure S28. ESI-HRMS of compound **4e**.

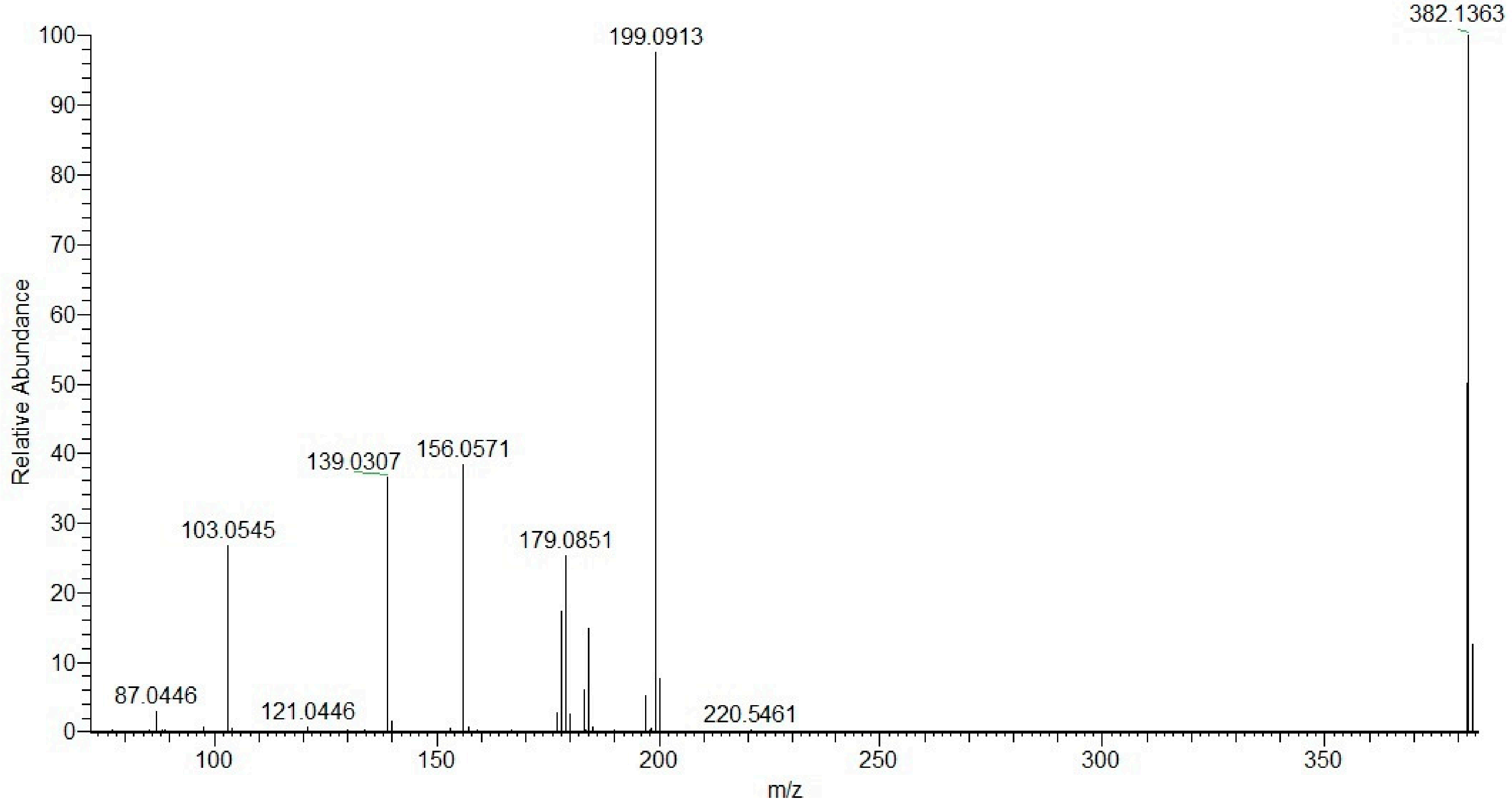

Figure S29. Mass spectrum of **4e** obtained by positive ion ESI-MS/MS.

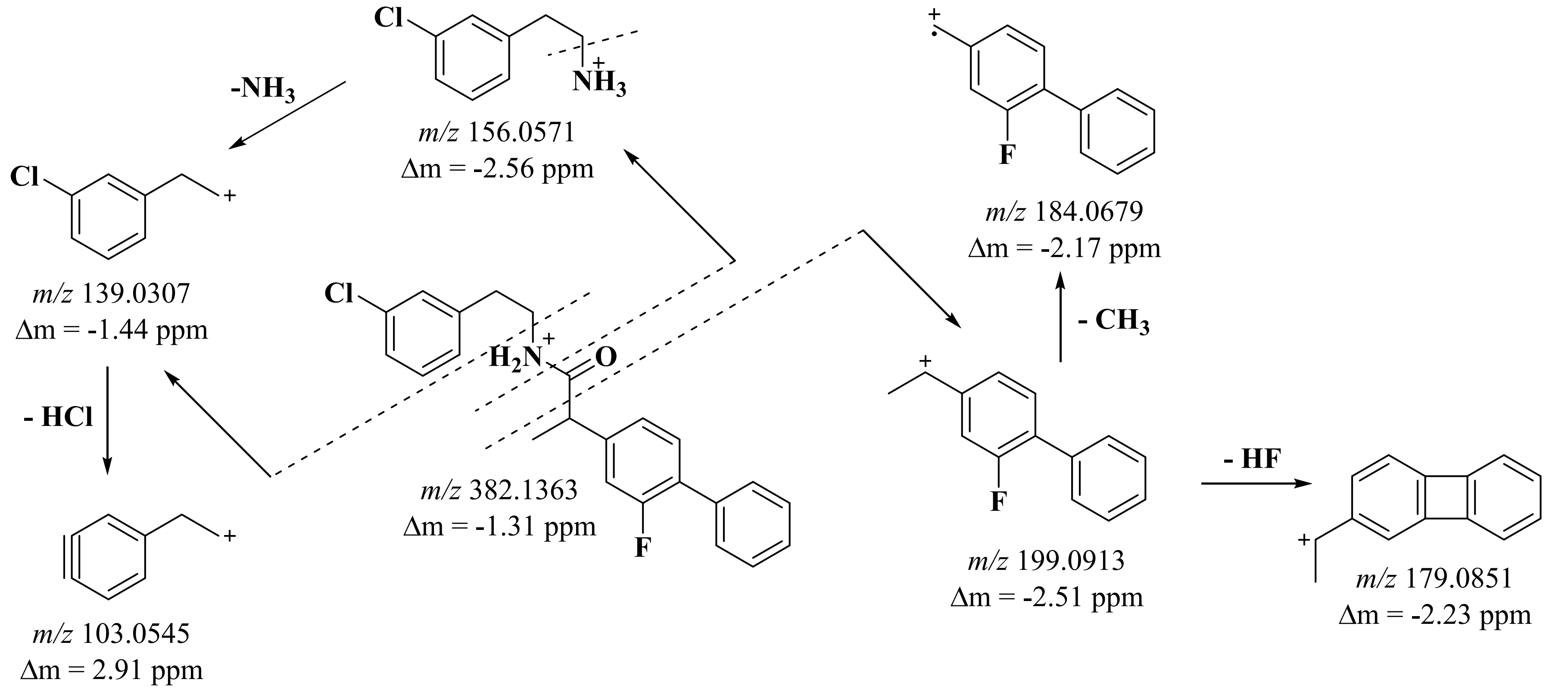

Figure S30. Proposed fragmentation of protonated **4e**.
